# Evaluation of different mucosal microbiota leads to gut microbiota-based prediction of type 1 diabetes in NOD mice

**DOI:** 10.1038/s41598-018-33571-z

**Published:** 2018-10-18

**Authors:** Youjia Hu, Jian Peng, Fangyong Li, F. Susan Wong, Li Wen

**Affiliations:** 1 Section of Endocrinology, Department of Internal Medicine, Yale University School of Medicine, New Haven, CT 06510 USA; 2 Yale Center for Analytical Sciences, Yale University School of Public Health, New Haven, CT 06510 USA; 3 Division of Infection and Immunity, School of Medicine, Cardiff University, Cardiff, UK

## Abstract

Type 1 diabetes (T1D) is a progressive autoimmune disease in which the insulin-producing beta cells are destroyed by auto-reactive T cells. Recent studies suggest that microbiota are closely associated with disease development. We studied gut, oral and vaginal microbiota longitudinally in non-obese diabetic (NOD) mice. We showed that the composition of microbiota is very different at the different mucosal sites and between young and adult mice. Gut microbiota are more diverse than oral or vaginal microbiota and the changes were more evident in the mice before and after onset of diabetes. Using alpha-diversity, Gram-positive/Gram-negative ratio as well as the relative abundance of *Bacteroidetes* and *Erysipelotrichaceae* in the gut microbiota, at 8 weeks of age, we formulated a predictive algorithm for T1D development in a cohort of 63 female NOD mice. Using this algorithm, we obtained 80% accuracy of prediction of diabetes onset, in two independent experiments, totaling 29 mice, with Area Under the Curve of 0.776 by ROC analysis. Interestingly, we did not find differences in peripheral blood mononuclear cells of the mice at 8 weeks of age, regardless of later diabetes development. Our results suggest that the algorithm could potentially be used in early prediction of future T1D development.

## Introduction

Type 1 diabetes (T1D) is a progressive autoimmune disease in which the insulin-producing beta cells in the pancreatic islets are destroyed by auto-reactive T cells. Many genes contribute to susceptibility to T1D, among which the most important susceptibility loci are those that code for Human Leukocyte Antigens (HLA). However, the rise in T1D incidence seen in recent years, especially in individuals who have lower risk HLA types^1^, cannot be explained by genetic changes, indicating that non-genetic factors influence disease development. Increasing evidence suggests that microbiota are closely associated with the development of T1D in both mouse models of human T1D and patients with T1D^2–7^.

The non-obese diabetic (NOD) mouse is an excellent animal model for studying pathogenesis of T1D as NOD mice develop spontaneous diabetes, with similarities to the disease development in humans^8^. Studies using NOD mice have made significant contributions to our understanding of immunopathogenesis, including the role of gut microbiota, in T1D development^9–14^. It is known that the gut microbiota provide nutrients to the hosts and coevolve with the host immune system. However, it is not clear when gut microbiota are altered to a composition that facilitates the autoimmune destruction of insulin-producing beta cells, which leads to T1D onset. In this study, we investigated dynamic changes of microbiota, from different anatomical sites in NOD mice, and their association with T1D development. We found that gut microbiota from NOD mice at 8 weeks of age underwent the most significant changes, some of which can be used to predict diabetes development later in life with 80% accuracy. Our study provides important information in assisting the prediction of T1D development and suggests that this approach could be used as a biomarker for T1D development. Our ultimate goal is to apply these biomarkers to finding more effective means to prevent T1D.

## Results

### Evolution of gut, oral and vaginal microbiota during maturation in NOD mice

To study the microbiota over time in NOD mice, we collected fecal, oral and vaginal samples from the training cohort of mice from week 3 to week 31 (details in Supplementary Fig. 1).

**Figure 1:**
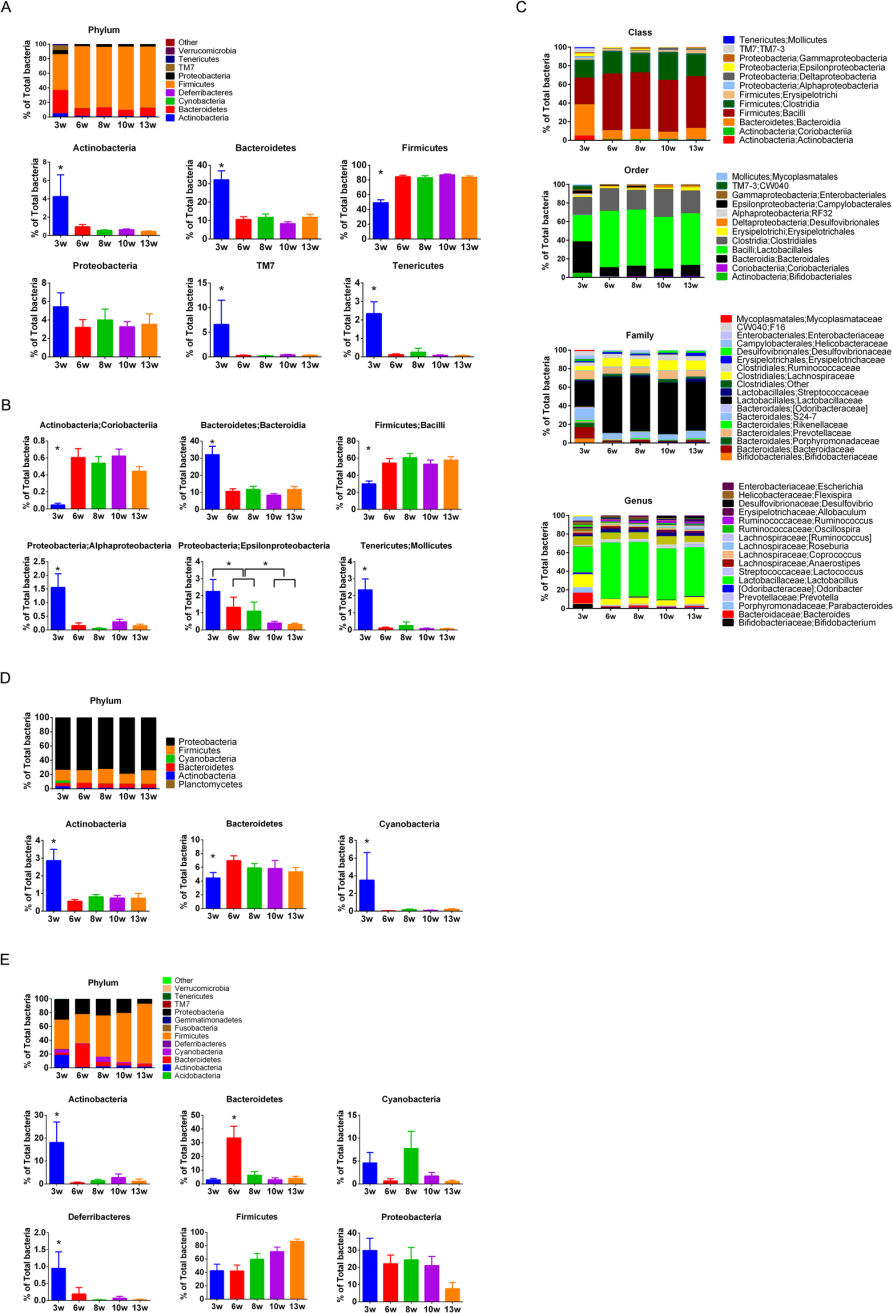
Fecal, oral and vaginal bacteria during maturation in NOD mice. ^*^Denotes statistical significance, p < 0.05. (**A**) Fecal bacterial composition at phylum level, and single phyla comparison between 3w, 6w, 8w, 10w and 13w (week-old) NOD mice. (**B**) Selected single class comparison between NOD mice of different ages. (**C**) Fecal bacterial composition at class, order, family and genus level between NOD mice at different ages. (**D**) Oral bacterial composition at phylum level, and selected single phyla comparison between NOD mice at different ages. (**E**) Vaginal bacterial composition at phylum level, and selected single phyla comparison between NOD mice at different ages.

First we analyzed the gut microbiota of the mice in the initial training cohort at 3 to 13 weeks. It is clear that the composition of gut microbiota at 3 weeks of age was significantly different from that at 6, 8, 10 and 13 weeks of age. These differences could be seen both at the phylum and class levels. The notable decrease in the individual phyla included *Actinobacteria, Bacteroidetes, Proteobacteria, TM7* and *Tenericutes*, whereas the phylum *Firmicutes* was increased after 3 weeks of age (Fig. 1A).

Several classes of gut bacteria also differed significantly with age. These included the increased *Coriobacteriia* and *Bacilli* classes of *Actinobacteria* and *Firmicutes* phyla, respectively, although the parental phylum *Actinobacteria* was decreased along with growth and maturation (Fig. 1B). The classes of *Bacteroidia, Alphaproteobacteria, Epsilonproteobacteria* and *Mollicutes*, which belong to phyla of *Bacteroidetes, Proteobacteria* and *Tenericutes*, respectively, were decreased (Fig. 1B). The differences were also observed at order, family, genus and species levels, with similar trends to their higher orders (Fig. 1C).

We also investigated the oral microbiota and did not find obvious differences between 3-week and older mice, as seen in the lower gut microbiota. Although the most abundant phylum in the oral cavity was *Proteobacteria*, we found a decrease in *Actinobacteria* and *Cyanobacteria* but an increase in *Bacteroidetes* (Fig. 1D).

Next, we analyzed the vaginal microbiota and observed a larger variation between young and adult mice and the composition changes were constant in some phyla from 3 to 13 weeks old. *Actinobacteria* and *Deferribacteres* were the highest at 3 weeks, *Bacteroidetes* were the highest at 6 weeks, while *Cyanobacteria* were the highest at 8 weeks of age. Interestingly, *Firmicutes* increased with maturation, whereas *Proteobacteria* decreased (Fig. 1E).

### Differences among gut, oral and vaginal microbiota in NOD mice during maturation

Comparing the three mucosal sites, the microbiota composition was very different between gut, oral and vaginal bacteria (Fig. 1). At a young age, *Proteobacteria* was the dominant phylum in the oral cavity (Fig. 1D), whereas *Firmicutes* was the dominant phylum of gut bacteria (Fig. 1A), while the composition of vaginal microbiota was intermediate between the two, although *Firmicutes* appeared to be more dominant (Fig. 1E). Gut and oral microbiota became relatively stable after 6 weeks of age, while vaginal microbiota continued to evolve until 10 to 13 weeks of age, by which time they resembled gut microbiota.

### Alpha-diversity of gut, oral and vaginal microbiota during maturation

**Figure 2:**
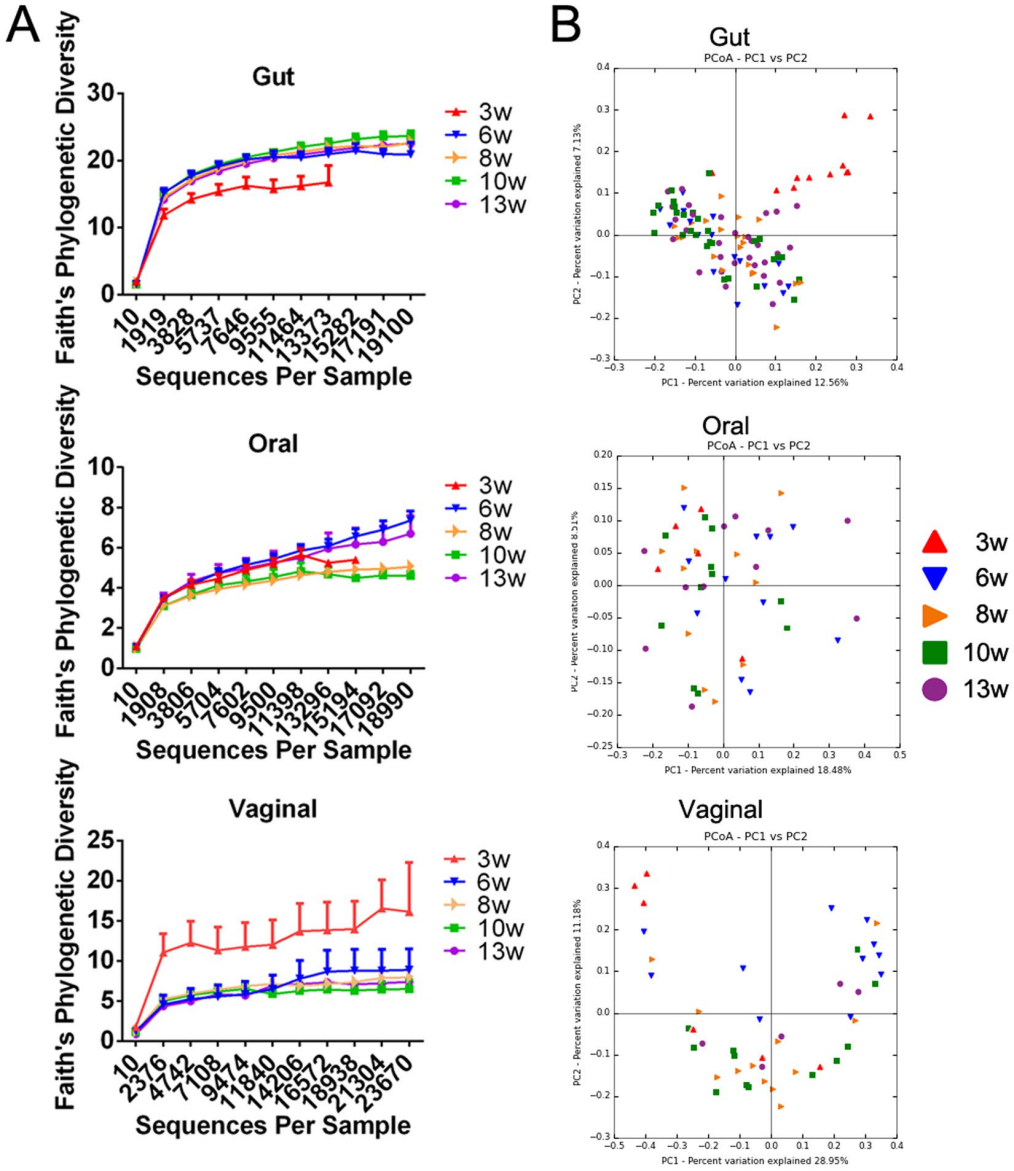
Comparison of gut, oral and vaginal microbiota during maturation in NOD mice. (**A**) Alpha-diversity of gut, oral and vaginal microbiota of NOD mice at different ages. (**B**) Beta-diversity of gut, oral and vaginal microbiota of NOD mice at different ages.

We next examined alpha-diversity of the gut/oral/vaginal microbiota, and found that the greatest diversity among the three anatomical sites was in the gut microbiota. Although at 3 weeks of age, gut microbiota were less diverse compared to the same mice at a later age, there was still greater diversity compared to the oral and vaginal microbiota from the same mice at any age. The changes in alpha-diversity of oral microbiota were similar during maturation, while vaginal microbiota showed the highest diversity when the mice were 3 weeks old and then gradually decreased, similar to the oral microbiota (Fig. 2A). This was opposite to the gut microbiota of the three-week-old mice.

### Beta-diversity of gut, oral and vaginal microbiota during maturation

Beta-diversity analysis showed that 3-week-old mice have different gut microbiota compared with the same mice at later time points. It is interesting that we did not find noticeable differences in beta-diversity of microbiota between the oral and vaginal sites regardless of the age (from 3w to 13w; Fig. 2B). This indicated that comparing the three mucosal sites, gut microbiota are the most dynamic during mouse maturation. The gut microbiota in the NOD female mice undergo significant changes from weaning (3 weeks) to maturation (8 weeks) and become stable thereafter. Although oral and vaginal microbiota also underwent some changes during this period, the differences were not significant.

### Diabetes incidence of NOD mice

While investigating the microbiota in the three anatomical sites, we also observed the experimental training cohort of mice for diabetes development. Of the 63 female NOD mice, 39 mice developed diabetes from 12 to 30 weeks of age. The overall incidence of diabetes was 61.9%, which was comparable to the diabetes incidence in our NOD mouse colony (a total of 144 females; Supplementary Fig. 2).

### Gut, oral and vaginal microbiota in non-diabetic and pre-diabetic NOD mice

**Figure 3:**
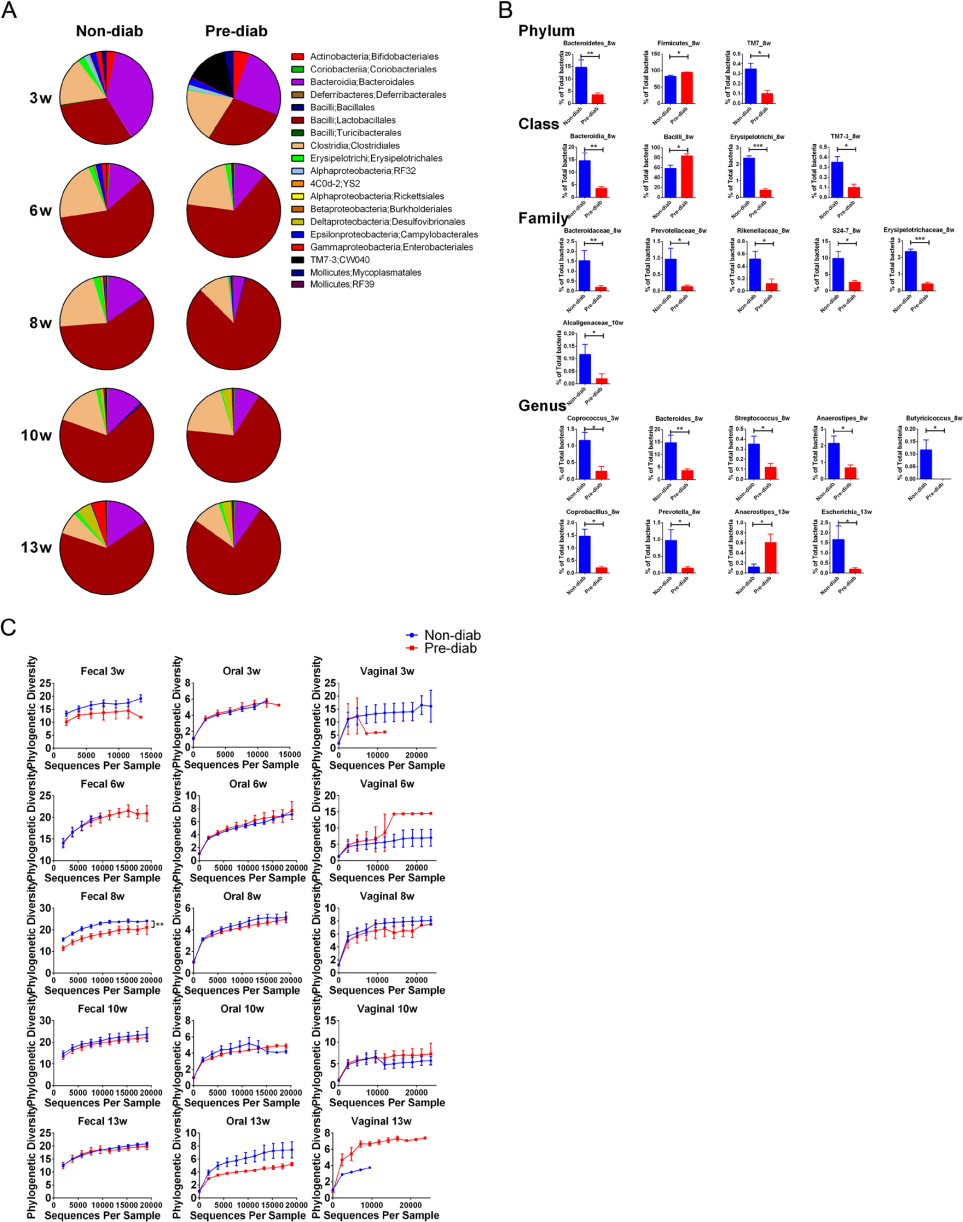
Comparison of gut microbiota between non-diab (mice that had not developed diabetes at the end of the observation period) and pre-diab (mice that later developed diabetes) NOD mice at different ages. (**A**) Gut bacterial composition at the order level is shown. (**B**) Selected single phyla, class, family and genus that show significantly different abundance between non-diab and pre-diab mice. Each figure title indicates the taxon and time point. ^*^P < 0.05, ^**^P < 0.01, ^***^P < 0.001 by multiple t test with Sidak-Bonferroni correction. (**C**) Alpha-diversity of gut, oral and vaginal microbiota between non-diab and pre-diab NOD mice at different ages. At week 8, non-diab and pre-diab NOD mice showed significantly different alpha-diversity in gut microbiota. ^**^P < 0.01 by two-way ANOVA.

Next, we compared the gut microbiota in fecal samples, at different taxonomic levels and different ages, from the mice that were non-diabetic at 31 weeks (unlikely to develop diabetes beyond this age based on extensive experience of studying our NOD mouse colony) and those that were pre-diabetic (mice that developed diabetes before 31 weeks but had not developed diabetes at the time of gut microbiota sampling). Although the overall composition was similar between 3 and 13 weeks of age (Fig. 3A), several taxa showed significant differences after the Sidak-Bonferroni correction and there were more differences at the family and genus levels (Fig. 3B). Importantly, most of the differences were observed at 8 and 10 weeks of age, indicating that the age of around 2 months is important for the establishment of gut microbiota and this also may be the critical time for predicting whether a NOD female mouse would later develop diabetes.

We found that the composition of oral microbiota between non-diabetic and pre-diabetic mice was similar; however, the composition of vaginal microbiota was very different between non-diabetic and pre-diabetic mice (Supplementary Fig. 3). Since we did not observe a significant correlation between the vaginal bacterial profile and diabetes onset, as the variation between individual mice was very large, we did not pursue this further.

### Alpha-diversity between non-diabetic and pre-diabetic NOD at different ages

We also compared the alpha-diversity between mice that were ultimately non-diabetic and pre-diabetic mice (these mice later developed diabetes) (Fig. 3C). At week 8, we found significantly greater alpha-diversity in the gut microbiota of the non-diabetic group compared with that of the pre-diabetic group. It is possible the changes at this age might have some impact on the host later in life, since this is the time when islet infiltration has begun but diabetes has not yet developed. In the oral and vaginal microbiota, there were no significant differences between the non-diabetic and the pre-diabetic groups, except at week 13. However, the overall diversity in the oral and vaginal microbiota was much lower than that in the gut microbiota.

### Beta-diversity between non-diabetic and pre-diabetic NOD at different ages

**Figure 4:**
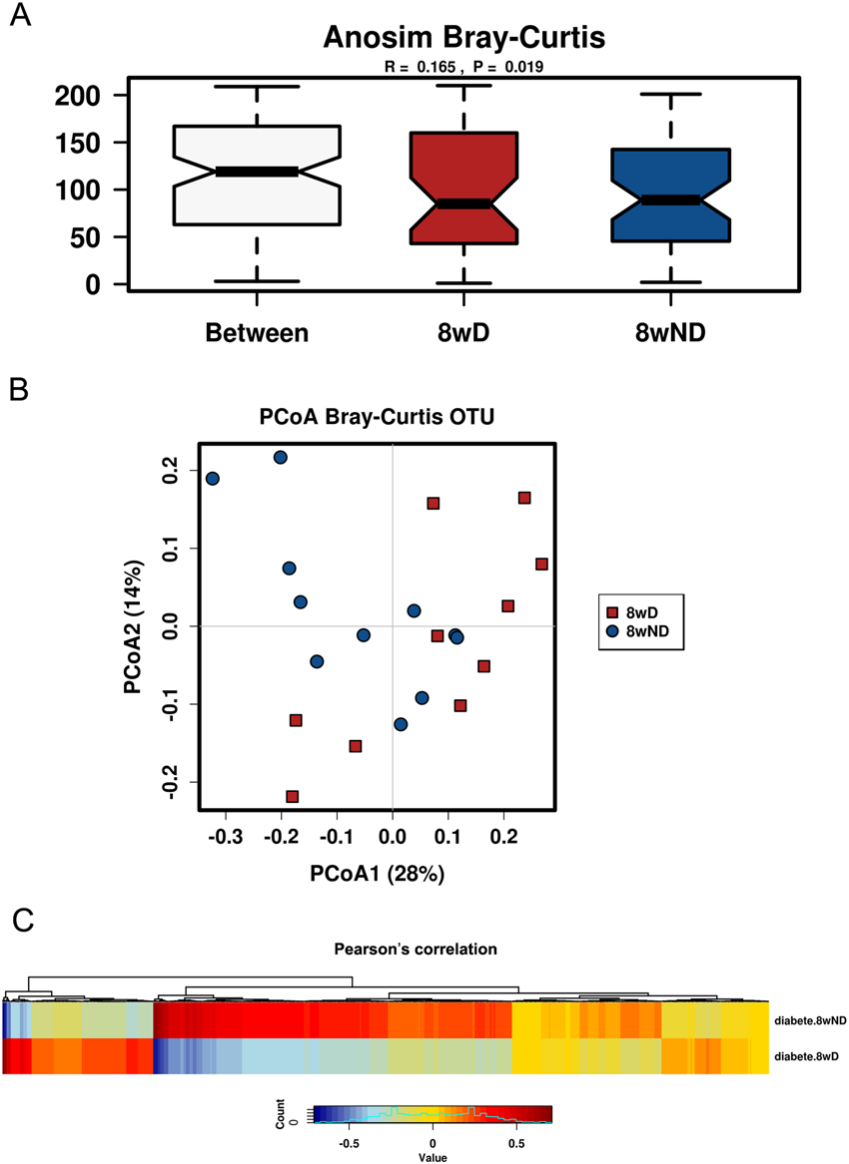
Multivariate testing using pre-diabetic (D) or non-diabetic (ND) and age as well as OTUs as variants analyzed by Calypso. (**A**) ANOSIM based on Bray-Curtis distance. (**B**) Principal Coordinate Analysis (PCoA) based on Bray-Curtis OTU. (**C**) Pearson’s correlation by OTU cluster.

By multivariate testing (ANOSIM), adding diabetic/non-diabetic and age as separation variants, we found at age of 8 weeks, the non-diabetic and pre-diabetic NOD mice showed significant differences in OTU composition based on Bray-Curtis distance (P = 0.019, Fig. 4A). PCoA analysis and Pearson’s correlation also showed some differences between these two groups at week 8 (Fig. 4B,C). However, there were no differences at weeks 3, 6, 10 and 13 (Supplementary Fig. 4), although there were some differences at week 3, but only by PCoA analysis (data not shown). From these results, we further concluded that the age of around two months is important for establishment of homeostasis of gut microbiota, and it may be possible to predict later diabetes development by gut microbiota at week 8.

### Gut microbiota evolution after mice become diabetic

We also studied the fecal samples collected from the mice after diabetes diagnosis (within 5 days) and found there was reduced alpha-diversity in the diabetic mice (Supplementary Fig. 5). It is interesting that the level of alpha-diversity in diabetic mice was similar to that seen in immature 3-week old mice. Moreover, the lower alpha-diversity was also observed in pre-diabetic mice at week 8 compared to the non-diabetic mice (Fig. 3C). However, the reduction in alpha-diversity was more evident after developing diabetes. Taxonomic changes were also different in non-diabetic and diabetic mice at the age of 16 to 27 weeks, comparing to the differences in non-diabetic and pre-diabetic mice at same ages, especially at the Genus level (Supplementary Fig. 6). Some of these alterations included *Coprobacillus*, *Staphylococcus, and Escherichia*, which are often reported to be pathogenic^15,16^, and were significantly increased (Supplementary Fig. 6).

### Altered Gram-positive (G+)/gram-negative (G−) ratio during maturation and with diabetes development

Among the eight major phyla, *Actinobacteria, Firmicutes, TM7* and *Tenericutes* are G+, while *Bacteroides, Cyanobacteria, Deferribacteres* and *Proteobacteria* are G−. Our results in the training cohort revealed that the ratio of G+to G− bacteria was significantly increased when the mice became diabetic. Importantly, the increased G+/G− ratio in diabetic mice was not associated with age of diabetes onset (Supplementary Fig. 7A). From week 3 to week 13, we also found an overall higher trend of G+/G− ratio in pre-diabetic NOD mice, while the most significant difference was seen at week 6 and 8 (Supplementary Fig. 7B). Thus, the G+/G− ratio could be used as a marker to predict whether an individual young mouse may eventually develop diabetes.

### Overall taxonomic changes of gut microbiota between non-diabetic and pre-diabetic NOD mice in the training cohort

Analyzing the overall taxonomic changes between non-diabetic and pre-diabetic mice at week 8 in the training cohort, we found decreased phyla *Bacteroidetes, Cyanobacteria, Proteobacteria, TM7* and *Tenericutes*, classes of *Coriobacteriia, Bacteroidia, 4C0d-2, Erysipelotrichi, Proteobacteria (*alpha/beta/delta/gamma), *TM7–3, Mollicutes* but increased classes of *Actinobacteria, Bacilli, Clostridia* and epsilon *Proteobacteria* (Supplementary Fig. 8). There were clear changes between non-diabetic and pre-diabetic mice in most of the families from the phyla *Bacteroidetes* and *Firmicutes*, especially the class of *Erysipelotrichi*. These changes are presented as a heatmap shown in Supplementary Fig. 9. There is a clear profile showing that the abundance of *Bacteroidetes* was decreased in the pre-diabetic mice. However, although the overall abundance of *Firmicutes* was increased in the pre-diabetic mice, the family of *Erysipelotrichaceae* showed the opposite profile, i.e., reduced in pre-diabetic mice (Supplementary Fig. 9). Since the phylum *Bacteroidetes* and family *Erysipelotrichaceae* were the most significantly different between non-diabetic and pre-diabetic groups, we chose these two taxa as indicators to predict whether our experimental test mice would eventually develop diabetes in the later studies.

In summary, our results show that alpha-diversity, G+/G− ratio and microbiota composition change (especially the phylum *Bacteroidetes* and Family *Erysipelotrichaceae*) may be used for prediction of future diabetes development in NOD mice at the age of 8 weeks. We next used each of the three indicators, as well as the three markers combined, to predict diabetes development in individual mice in the subsequent test experiments.

### Using alpha-diversity for prediction of diabetes development

**Figure 5:**
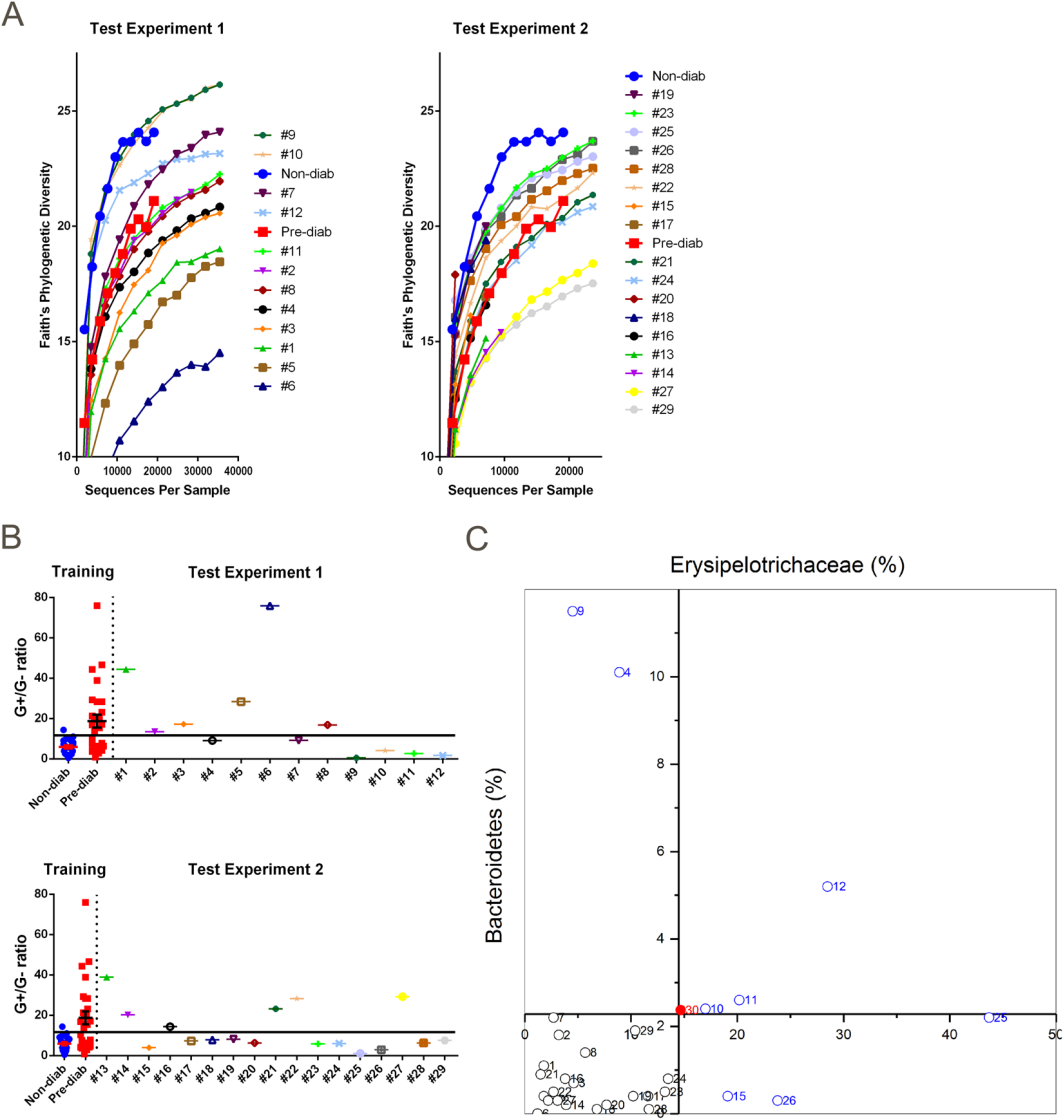
Prediction of diabetes development in NOD mice at 8 weeks of age. The prediction experiment was repeated using two test groups, comprising 12 and 17 mice, respectively. (**A**) Use of gut bacterial alpha-diversity for prediction of diabetes development. The individual mouse numbers in the figure are listed in the order of the mouse that had the highest diversity of gut bacteria to the lowest diversity. Non-diab (filled blue circles) and pre-diab (filled red squares) represent the average diversity of the bacteria from non-diab mice and pre-diab mice, respectively, from the training cohorts used as the reference points for comparison with the test experiment. Mice with gut bacterial alpha diversity higher than pre-diab were predicted not to develop diabetes by the end of observation (i.e., mice 9, 10, 7 and 12 in experiment 1 and 19, 23, 25, 26, 28, 22, 15 and 17 in experiment 2). (**B**) Use of gut bacterial G+/G− ratio for diabetes prediction. The cut-off line was drawn at 15.3 to separate the non-diabetic and pre-diabetic groups according to ROC analysis. Non-diab and pre-diab were the gut bacterial G+/G− ratio of non-diab mice and pre-diab mice, respectively, from the training cohorts. G+/G− ratio of single experimental mice were plotted and the mouse was predicted to be non-diab if the ratio was below the cut-off line or pre-diab if the ratio was above the cut-off line. (**C**) Use of gut taxonomic profile for prediction. The abundance of Phylum *Bacteroidetes* and Family *Erysipelotrichaceae* of the 29 experimental mice were plotted on this 2D scatter diagram. #30 (the red solid circle) represents the average abundance of *Bacteroidetes* and *Erysipelotrichaceae* of all non-diabetic NOD mice in the training cohort.

We carried out two independent test experiments, with 12 and 17 female NOD mice in each test cohort and predicted diabetes development using the sequencing data of their gut microbiota at the age of 8 weeks. We compared the gut bacterial alpha-diversity of the mice from the two independent experiments to the average gut bacterial alpha-diversity from the training cohort. As there was a significantly higher gut bacterial alpha-diversity in the non-diabetic mice when they were 8 weeks old in the training cohort, we plotted the gut bacterial alpha-diversity of each individual mouse in the test cohort, and compared to the average gut bacterial alpha-diversity of pre-diabetic and non-diabetes group in the training cohort (Fig. 5A). In both experimental groups, some mice showed the gut bacterial alpha-diversity close to or lower than the pre-diabetic mice in the training cohort (red line) whereas others showed gut bacterial alpha-diversity close to or higher than the average gut bacterial alpha-diversity of non-diabetic mice in the training cohort (blue line) (Fig. 5A). We predicted that the mice having gut bacterial alpha-diversity below the red line would develop diabetes, whereas the mice that had gut bacterial alpha-diversity above it would not develop diabetes (predicted result and the actual observation result are listed in Table 1). The accuracy of our prediction was 69% (20 out of 29 mice had the correct diabetes or non-diabetes prediction) if gut bacterial alpha-diversity was used as the sole prediction marker.

**Table 1:** Diabetes prediction of NOD mice at week 8 of age according to gut microbiota.

#	Alpha-diversity	G+/G− ratio	Taxa	Combined	Observation
1	***Diabetic***	***Diabetic***	***Diabetic***	***Diabetic***	Diabetic
2	***Diabetic***	***Diabetic***	***Diabetic***	***Diabetic***	Diabetic
3	***Diabetic***	***Diabetic***	***Diabetic***	***Diabetic***	Diabetic
4	Diabetic	***Non-diab***	***Non-diab***	***Non-diab***	Non-diab
5	***Diabetic***	***Diabetic***	***Diabetic***	***Diabetic***	Diabetic
6	***Diabetic***	***Diabetic***	***Diabetic***	***Diabetic***	Diabetic
7	***Non-diab***	***Non-diab***	Diabetic	***Non-diab***	Non-diab
8	***Diabetic***	***Diabetic***	***Diabetic***	***Diabetic***	Diabetic
9	***Non-diab***	***Non-diab***	***Non-diab***	***Non-diab***	Non-diab
10	Non-diab	Non-diab	Non-diab	Non-diab	Diabetic
11	Diabetic	***Non-diab***	***Non-diab***	***Non-diab***	Non-diab
12	***Non-diab***	***Non-diab***	***Non-diab***	***Non-diab***	Non-diab
13	***Diabetic***	***Diabetic***	***Diabetic***	***Diabetic***	Diabetic
14	***Diabetic***	***Diabetic***	***Diabetic***	***Diabetic***	Diabetic
15	***Non-diab***	***Non-diab***	***Non-diab***	***Non-diab***	Non-diab
16	Diabetic	Diabetic	Diabetic	Diabetic	Non-diab
17	***Non-diab***	***Non-diab***	Diabetic	***Non-diab***	Non-diab
18	***Diabetic***	Non-diab	***Diabetic***	***Diabetic***	Diabetic
19	Non-diab	Non-diab	***Diabetic***	Non-diab	Diabetic
20	***Diabetic***	Non-diab	***Diabetic***	***Diabetic***	Diabetic
21	***Diabetic***	***Diabetic***	***Diabetic***	***Diabetic***	Diabetic
22	Non-diab	***Diabetic***	***Diabetic***	***Diabetic***	Diabetic
23	***Non-diab***	***Non-diab***	Diabetic	***Non-diab***	Non-diab
24	***Diabetic***	Non-diab	***Diabetic***	***Diabetic***	Diabetic
25	Non-diab	Non-diab	Non-diab	Non-diab	Diabetic
26	Non-diab	Non-diab	Non-diab	Non-diab	Diabetic
27	***Diabetic***	***Diabetic***	***Diabetic***	***Diabetic***	Diabetic
28	***Non-diab***	***Non-diab***	Diabetic	***Non-diab***	Non-diab
29	Diabetic	***Non-diab***	Diabetic	Diabetic	Non-diab

(Bold italic shows that the prediction is the same with the observation).

### Using G+/G− ratio to predict diabetes development

To test whether G+/G− ratio could also be used as a marker to predict diabetes development, we calculated gut bacterial G+/G− ratio of all the mice used in the training cohort at 8 weeks of age (P = 0.001 by unpaired t-test comparing non-diabetic and pre-diabetic mice) and used the calculated value to predict whether the mice in the two test cohorts would develop diabetes. The optimal cut-off value at 15.3 achieved 100% sensitivity and 57% specificity to discriminate the pre-diabetic mice from non-diabetic ones according to ROC analysis. The gut bacterial G+/G− ratios of the individual mice in the test groups are shown in Fig. 5B, with the prediction and actual diabetes observation result are listed in Table 1. The accuracy of our prediction was 72% if using the gut bacterial G+/G− as the sole marker.

### Using microbiota composition change to predict diabetes development

We plotted the percentage of *Bacteroidetes* and *Erysipelotrichaceae* in a 2D scatter graph. As the abundance of these two taxa was significantly altered in pre-diabetic mice, we proposed that the mice having a lower abundance of *Bacteroidetes* and *Erysipelotrichaceae* may later develop diabetes (lower left quadrant, Fig. 5C), compared to the average abundance of these two taxa in the non-diabetic mice from the training cohort. Table 1 lists the results from our prediction and the results from the actual observation. The accuracy of our prediction was 69% if using the percentage of *Bacteroidetes* and *Erysipelotrichaceae* as the only marker.

### Prediction of diabetes development using the combined indicators

**Figure 6:**
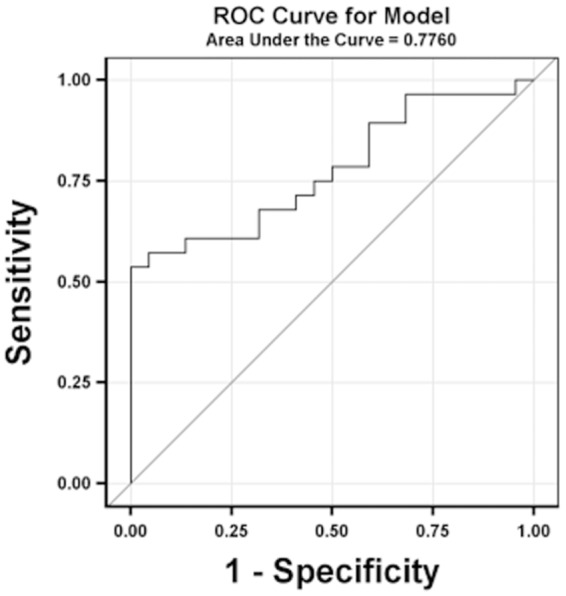
ROC analysis of sensitivity and specificity of the three-indicator-model for prediction of diabetes development in NOD mice at 8 weeks of age.

Finally, we used all three sets of data together to predict the diabetes development of the 29 experimental mice in the two independent experimental test cohorts, and monitored glycosuria for diabetes development weekly to 31 weeks (Table 1). If 2 or 3 of the three parameters indicated diabetic (or non-diabetic), we predicted the mouse would have (or not) developed diabetes. Thus, we predicted 16 mice would have developed diabetes (14 mice did develop diabetes later and 2 did not) and 13 mice would have not developed diabetes (9 mice did not develop diabetes by the time the experiments ended but 4 developed diabetes during the observation period). Thus, using the combined parameters, we were able to achieve ~80% predictive accuracy (23 out of 29 mice had the correct prediction) (Table 1). We also performed ROC analysis to test the sensitivity and specificity of the parameters used for diabetes prediction (Fig 6 and Table 2), with area under the curve (AUC) of 0.776. Gut bacterial G+/G− ratios or taxa alone gave the highest specificity or sensitivity, respectively. However, prediction utilizing all three parameters gave overall higher sensitivity, specificity, positive and negative predictive values (Table 2).

**Table 2:** ROC analysis of diabetes prediction using different indicators.

	Alpha-diversity	G+/G− ratio	Taxa	Model t
Sensitivity	0.72 (0.47, 0.90)	0.61 (0.36, 0.83)	0.83 (0.59, 0.96)	0.78 (0.52, 0.94)
Specificity	0.64 (0.31, 0.89)	0.91 (0.59, 1.00)	0.45 (0.17, 0.77)	0.82 (0.48, 0.98)
Positive predictive value	0.76 (0.50, 0.93)	0.92 (0.62, 1.00)	0.71 (0.48, 0.89)	0.88 (0.62, 0.98)
Negative predictive value	0.58 (0.28, 0.85)	0.59 (0.33, 0.82)	0.63 (0.24, 0.92)	0.69 (0.39, 0.91)

(Each column represents calculated value (confidence intervals)).

### Immune cell characterization

To identify whether there were changes in immune cells correlating with the subsequent development of diabetes, as shown for the gut microbiota, we characterized the immune cells in the peripheral blood of the mice when 8 weeks old, by flow cytometry. All the mice tested showed similar frequencies of T cells (using anti-mouse CD4+ and CD8+ antibodies), B cells (using anti-mouse B220/CD19 antibodies), macrophages (using anti-mouse CD11b antibody), dendritic cells (using anti-mouse CD11c antibody) and the cytokine profiles of the immune cells (using anti-mouse IL-10/IL-17/IFN-γ antibodies) were also similar in all the mice tested (Supplementary Fig. 10). Furthermore, the co-stimulatory marker expression (using anti-mouse CD80 antibody), Treg (defined by gated CD4+CD25+FoxP3+ cells), Breg (defined by gated CD1d^high^CD5+B220+CD19+ cells) and transitional B cells (defined by gated CD21+CD24+B220+CD19+ cells) were also similar between non-diabetic and diabetic groups (Supplementary Fig. 10). Our results showed that at the early time point of 8 weeks of age in NOD mice, there were no distinct immune cell features in the peripheral blood that correlated with marked differences in microbiota, comparing those mice that were ultimately non-diabetic with those that were pre-diabetic mice. Our data also suggested that the alteration of gut microbiota is likely to be an early biomarker for prediction of T1D development.

## Discussion

In this study we first investigated the gut, oral and vaginal microbiota in 63 female NOD mice longitudinally. Among the three anatomical sites, gut microbiota presented the most consistent and significant changes with age and diabetes development. Using the analytical results from the gut microbiota, especially at the age of 8 weeks, we retrospectively examined the association with diabetes development in this cohort. We found that 3 separate measures - alpha-diversity, G+/G− ratio and some specific taxonomic differences were closely associated with diabetes development. We then tested the three parameters in two independent prospective studies and found that these parameters could predict diabetes development with 80% accuracy (78% sensitivity and 82% specificity) when the mice were only 8 weeks old, an age when there is no detectable immune cell alteration in the peripheral blood but immune infiltration of the pancreas could be present. Although *Tbk1*, *Psmc2* and *Dag1* expressed in lymphocytes at week 8 to 14 have been shown to be negatively correlated with later diabetes development in NOD.CD45.2 mice^17^, this has not yet been validated by another experimental cohort. Here, our study uses fecal microbiota to predict the diabetes development, and more notably, this study is conducted in wild type NOD mice, rather than knock-out or antibiotic-treated mice. Thus, these parameters could potentially be used as a novel biomarker for prediction of type 1 diabetes onset under normal circumstances. Our ultimate goal is to translate the finding from the current study to humans.

Like humans, many factors can influence the composition of the microbiota in NOD mice including housing conditions^18^, diet^19^ and pH of drinking water^20^. It is conceivable that the variations in gut microbiota in different NOD colonies around the world^21^ contribute to the different incidence of diabetes observed^22^. A newly published study showed that T1D susceptibility alleles can alter the gut microbiota^23^. In our colony, we found that the microbiota from two mucosal sites - gut (fecal), oral cavity had low diversity, but the microbiota from vagina had high diversity at 3 weeks of age, a feature of an “immature” microbiome community, compared to the microbiota from the same mucosal site at an older age. The oral and fecal microbiota appear to be stabilized from 6 to 13 weeks, the period of developing insulitis and pre-diabetes stage. Our results showed that 8 weeks of age is an important time window, when the composition of gut microbiota is closely associated with disease susceptibility and developing changes, leading to diabetes. We have identified several taxa within the phyla *Bacteriodetes*, *Firmicutes* and *Proteobacteria* that are strongly correlated with T1D development. Another study, analyzing the gut microbiota in the fecal samples collected at weaning from non-diabetic and pre-diabetic NOD mice, also revealed that the abundance of S24-7 family and *Prevotella* genus from phylum Bacteroidetes were significantly increased in the non-diabetic group^24^. Our results are consistent with this; however, we believe that more than one taxon is responsible for the development of the disease. In contrast, Bacteroidetes are increased in oral samples when mice become older. However, the most abundant taxon in the oral microbiota is the non-anaerobic Proteobacteria. Comparing to fecal and oral microbiota, vaginal microbiota are not as stable as the other two. This may be due to the variation in the estrous cycle at the time of sample collection, and more exposure to the environment, compared to gut microbiota. Consistent with previous publications^25,26^, we found that *Lactobacilli* were the major taxon in the vaginal microbiota. We suggest that T1D development is related to the homeostasis within the gut microbiota, as we also found alpha-diversity and G+/G− ratio are strongly correlated with disease development. This is likely to be tied in with the role of the microbiota in promoting the development of immune system^27^. We and others have shown that gut microbiota at the neonatal stage of development is critical to the host immune system, especially for the development and function of antigen presenting cells (APCs)^13,14,28^. One report showed that administration of *Clostridiales*, which are lacking in neonatal mice, can protect the mice from specific pathogen infection, while administration of *Bacteroidales* cannot^29^. This may also indicate the importance of gut microbiota homeostasis but not *Clostridiales* itself. Another example comes from mono-colonization of germ-free mice with a *Bacteroides* species that were shown to have resistance to colonization with the same species but could be colonized with a different species^30^.

Although individual bacteria such as segmented filamentous bacteria (SFB) induce Th17 cells, the effect requires the presence of other bacterial strains^31^. SFB have also been linked to protection from diabetes in NOD mice^32^ and the protection appears to occur only in male NOD mice and in the presence of other bacterial strains^33^. However, changes in SFB have not been the mechanism of protection in other models of T1D^34^, nor in our NOD colony (data not shown). We have recently found *Leptotrichia goodfellowii*, a member of the phylum *Fusobacteria*, promotes T1D onset in a T cell receptor transgenic NOD mouse model, where T cells are specific for an islet autoantigen, islet-specific glucose-6-phosphatase catalytic subunit related protein (IGRP)^11^. Interestingly, an increased abundance of these bacteria is also found in non-transgenic diabetic NOD mice^11^. However, it is unlikely that any single bacterial strain or species will be solely “pathogenic” or “protective”, as the gut microbiota make up a complex ecosystem with symbiotic and non-symbiotic mutualistic relationships. Genetics may play a role here, as shown in a recent report indicating that gut microbiota in NOD mice carrying protective alleles at T1D susceptibility loci, were shaped differently from the NOD mice without the protective alleles^23^. The abundance of *Lactobacillus* and S24-7, as well as *Bacteroides*, *Parabacteroides*, *Prevotella* and 5-7N15 in NOD mice, and *Bacteroides*, *Lachnospiraceae* and *Ruminococcaceae* in human were altered. However, it should be noted that some differences in bacteria could be causal but others could occur as a consequence of disease development.

The role of *Erysipelotrichales*, a family member of *Firmicutes*, in health and disease is poorly understood thus far. We have found in this study that *Erysipelotrichales* is closely associated with the susceptibility to T1D onset and it is the only family within the phylum *Firmicutes* found to be decreased in pre-diabetic mice at week 8, while the others were increased. Therefore, *Erysipelotrichales* abundance level can be used as an indicator to distinguish the non-diabetic and pre-diabetic NOD mice. Interestingly, the decreased abundance of *Erysipelotrichales* was strongly associated with disease status in treatment “naïve” patients with new-onset of Crohn’s disease^35^. Crohn’s disease is also an autoimmune disorder, and although the etiology and pathogenesis is different from T1D, the common feature of decreased *Erysipelotrichales* associating with disease onset is intriguing. Further investigation is needed to find the molecular mechanism(s).

*Firmicutes* and *Bacteroidetes* are two major phyla of gut microbiota and the ratio of *Firmicutes/Bacteroidetes* has been used as an indicator for T1D onset^36^, in both streptozotocin-induced^37^ and spontaneous diabetic NOD mice^38^, as well as children with T1D susceptibility^39^. We found that diabetic mice in our NOD colony have increased *Firmicutes* (mostly G+) and decreased *Bacteroidetes* (mostly G−) (data not shown), which is consistent with other studies in NOD mice^40^ and in T1D patients^41^. Using G+/G− ratio, as one of the indicators, we can predict T1D onset with 72% accuracy in the current study. Our results suggest that G+/G− ratio could be used as an additional biomarker for more accurate prediction of T1D onset, which in turn could assist in designing a better and/or more effective strategy for T1D prevention.

Increasing evidence suggests that metabolites produced by gut microbiota regulate the metabolic and immunological function. Short chain fatty acids (SCFAs) produced by gut microbiota have been shown to regulate the host immune system including T follicular helper cells^42^, T regulatory cells^43^ and gamma-delta T-17 cells^44^. Feeding NOD mice with acetate- or butyrate-enriched diets can influence the frequency of autoreactive T cells and modulate the susceptibility to T1D development in NOD mice^40^. It is, currently, not clear what metabolites are produced in *Erysipelotrichales* and other altered gut microbiota that affect diabetes development in our study. It is clear that T1D is a complex autoimmune disease and how the autoimmune reactivity against pancreatic-beta cells is initiated is not known. Different gut bacteria can induce different immune responses mediated by different components of the bacteria including lipopolysaccharides (LPS)^45^, peptidoglycan (PGN)^46^ and polysaccharide A (PSA)^47^. Vatanen and colleagues have recently reported that the different structure of LPS produced by *E. coli* in the gut contributes to T1D susceptibility in children, due to its differences in immunogenicity to the hosts^48^. Furthermore, gut microbiota altered by antibiotic treatment can affect the function of antigen presenting cells, which in turn promotes different immune responses in the hosts and regulates T1D susceptibility^13,14^.

In this study, we provide the first longitudinal profile of microbiota from three mucosal sites during NOD mouse maturation and after diabetes onset. Using alpha-diversity, G+/G− ratio and specific taxonomic changes in gut microbiota at 8 weeks of age, we are able to predict diabetes onset later in life in individual NOD mouse with 80% accuracy. This suggests that gut microbiota are very important early in life, especially during maturation. Many factors could contribute to the ~20% of NOD mice in which diabetes was not predicted using our model, which include but are not limited to viruses^49^, bacteriophage^50^ and fungi^51^ in the ecosystem of gut microbiota. Regardless, our results provide important and novel information, and also a promising biomarker as a predictive tool in early diagnosis of T1D. A future extension to our study would be to develop similar predictive markers, readily translatable to humans.

## Methods

### Mice

Female NOD/Caj mice were originally obtained from the Jackson Laboratory and have been maintained at Yale University for about 30 years. The mice used in this study were housed in specific pathogen-free (SPF) conditions with a 12-hour dark/light cycle, in individually-ventilated filter cages with autoclaved Rodent Diet 2018S (Envigo), bedding, and hyper-chlorinated filtered water, in the Yale University animal facility. The use of the animals and all experimental protocols in this study were approved by the Yale University Institutional Animal Care and Use Committee, and all experimental methods were performed in accordance with the relevant guidelines and regulations.

### Sample collection

Fecal, oral and vaginal samples were taken sequentially from our NOD mouse colony as shown in Supplementary Fig. 1 over a period of a year from 63 female mice, the offspring of over 20 different breeding pairs. Fecal samples were collected from fresh fecal pellets. Oral and vaginal samples were obtained using sterile cotton swabs to swab the specific sites. Samples were collected from the 63 mice, following the schedule indicated in Supplementary Fig. 1 until 31 weeks of age or until the mice became diabetic. We designated this collection as the training or discovery cohort. The samples from these mice were used to generate the data set that would form the basis for prediction of diabetes in the subsequent two independent sets of experimental mice – the test cohorts. From the test cohorts, totaling 29 experimental mice, fecal and blood samples were collected at 8 weeks of age, and they were subsequently observed for T1D development by screening urine weekly for glycosuria. Diabetes was then confirmed by blood glucose (≥250 mg/dl).

### Flow cytometry

Blood was taken into heparin-coated capillary tubes and added to 1 ml PBS immediately. Peripheral blood mononuclear cells (PBMCs) were isolated on 1 ml Lymphoprep (Stemcell) gradient density centrifugation according to the manufacturer’s protocol. After isolation, the PBMCs were washed with PBS and stained with pre-titrated monoclonal antibodies (mAbs) for cell-specific markers on T cells, B cells, macrophages and dendritic cells (Biolegend). For all the intracellular cytokine (ICC) staining, the cells were all cultured for 4 h with 50 ng/ml PMA (Sigma), 500 ng/ml of ionomycin (Sigma) and 1 mg/ml of Golgi plug (BD Bioscience), before staining with mAbs against surface markers including Fc blocker (Biolegend). Following fixation and permeabilization, the cells were then stained with pre-titrated anti-cytokine mAbs (Biolegend) and their expression was analyzed by flow cytometry.

### DNA extraction and 16S rRNA sequencing

Bacterial DNA from mouse feces, the oral cavity and vaginal samples was isolated as previously described^52^. The V4 region of the bacterial 16S ribosomal gene was amplified from each DNA sample using a barcoded, broadly conserved, bacterial forward primer (5′-GTGCCAGCMGCCGCGGTAA-3′) and reverse primer (5′-GGACTACHVGGGTWTCTAAT-3′). The PCR products were purified with a Qiagen gel extraction kit. After quantification of DNA concentration using the Qubit dsDNA HS assay kit, equimolar amounts of each sample were pooled and used for pyrosequencing. Sequencing was performed on the Ion Torrent Personal Genome Machine (PGM) sequencing system (Life Technologies) using 200 bp read chemistry.

### Sequence analysis

The sequencing results were analyzed using the QIIME software package (version 1.8) and UPA RSE pipeline (version 7.0). After removing the primer sequences, the sequences were demultiplexed, quality-filtered using QIIME, and further quality and chimera filtered in UPARSE pipeline. Operational taxonomic units (OTU) were picked with 97% identity in UPARSE pipeline. In QIIME, the Greengenes reference database (version 13.5) was used for taxonomy assignment, which was performed at various levels using representative sequences of each operational taxonomic unit. Beta-Diversity was calculated to compare differences between microbial community profiles, and the data are shown as a principal coordinate analysis (PCA). Multivariate testing was performed in Calypso (Version 8.62) by ANOSIM analysis. Alpha-diversity was measured by Faith’s Phylogenetic Diversity (PD_Whole_Tree) in the bacterial communities.

G+/G− ratio was calculated using Gram-positive (G+) bacteria (*Tenericutes*, *TM7*, *Firmicutes*, *Actinobacteria*) contents divided by Gram-negative (G−) bacteria (*Proteobacteria*, *Cyanobacteriia*, *Bacteroidetes*, *Deferribacteres*) contents at phylum level of sequencing results.

### Biostatistics

Statistical analyses were performed in GraphPad Prism (version 7). Two-way ANOVA and multiple t tests with Sidak-Bonferroni correction were used while p < 0.05 was considered to be significantly different. Receiver operating characteristic (ROC) analysis was performed using logistic regression method^53,54^. The area under curve (AUC) was used as index of accuracy of prediction. Youden index (J = max[sensitivity + specificity − 1]) was used to determine the optimal cut-off of G+/G− ratio.

## Electronic Supplementary Material

Supplementary Figures

## Data Availability

The datasets generated during and/or analysed during the current study are available from the corresponding author on reasonable request.
