# Supplementary material for: Evaluation of different mucosal microbiota leads to gut microbiota-based prediction of type 1 diabetes in NOD mice
Source: Sci Rep. 2018 Oct 18;8:15451. doi: 10.1038/s41598-018-33571-z (PMC6193974; doi:10.1038/s41598-018-33571-z)
Supplement: Supplementary file 1 — Supplementary Figures [file 41598_2018_33571_MOESM1_ESM.pdf]

Evaluation of different mucosal microbiota leads to gut microbiota-based prediction of type 1 diabetes in NOD mice

Youjia Hu[1], Jian Peng[1], Fangyong Li[2], F. Susan Wong[3] and Li Wen[1]*

[1]Section of Endocrinology, Department of Internal Medicine, Yale University School of Medicine, New Haven, CT 06510

[2]Yale Center for Analytical Sciences, Yale University School of Public Health, New Haven, CT 06510

[3]Division of Infection and Immunity, School of Medicine, Cardiff University, Cardiff, UK

*Corresponding author: Li Wen

S141 TAC, 300 Cedar Street, Yale University, New Haven, CT 06520

Tel: 203-785-7186, Email: li.wen@yale.edu

Supplementary Figure 1

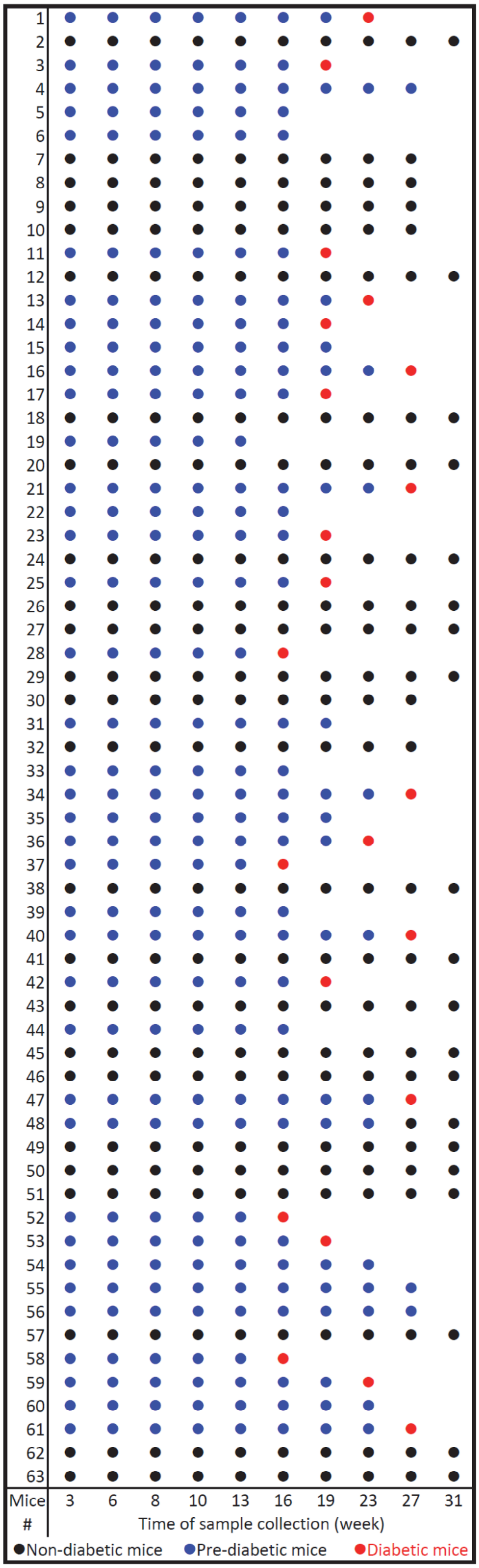

Supplementary Figure 1: Sample collection chart for observation group (training cohort). Fecal, oral and vaginal samples were collected at weeks 3,6,8,10,13,16,19,23,27,31 when available. Mice were screened for diabetes every week. Black dots (non-diab) represent the mice that had not developed diabetes by the end of the observation period (31 wks of age). Blue dots represent the mice at the pre-diabetic stage that had not developed diabetes (pre-diab) at the time of sample collection but became diabetic afterwards. Red dots represent the time point after the pre-diab mice became diabetic (diab).

Supplementary Figure 2

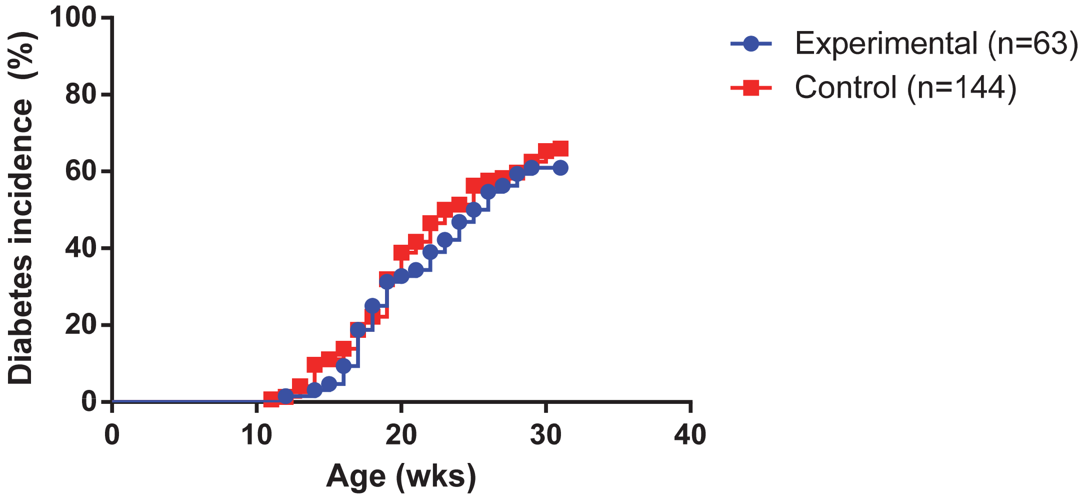

Supplementary Figure 2: Diabetes incidence in NOD mice that were included in the study observation group and control NOD mice in our NOD colony in the same year that were not included in the observation group.

Supplementary Figure 3A

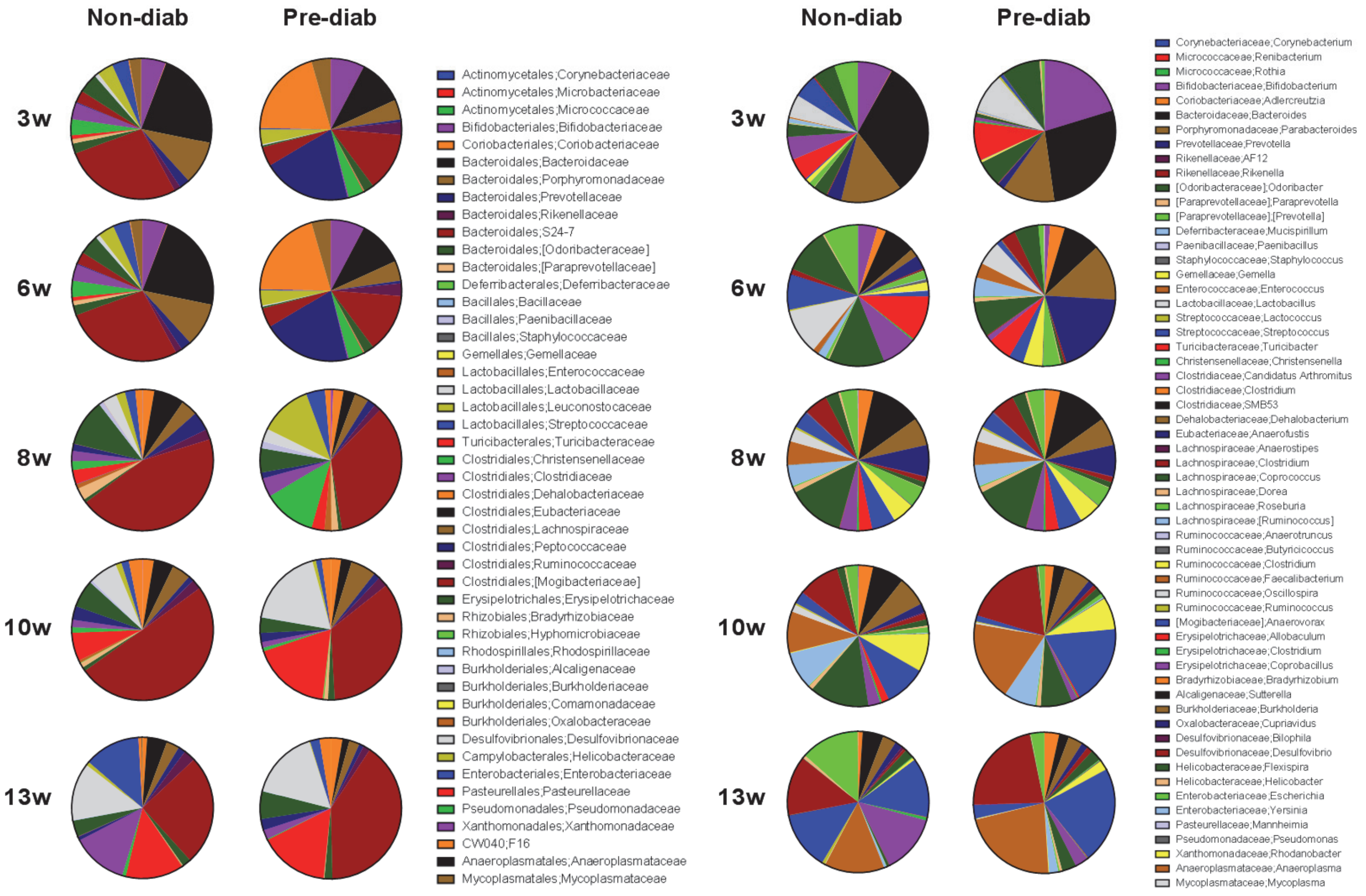

Supplementary Figure 3A: Gut microbiota at family (left two groups) and genus level (right two groups), in non-diab and pre-diab NOD mice at different ages.

Supplementary Figure 3B

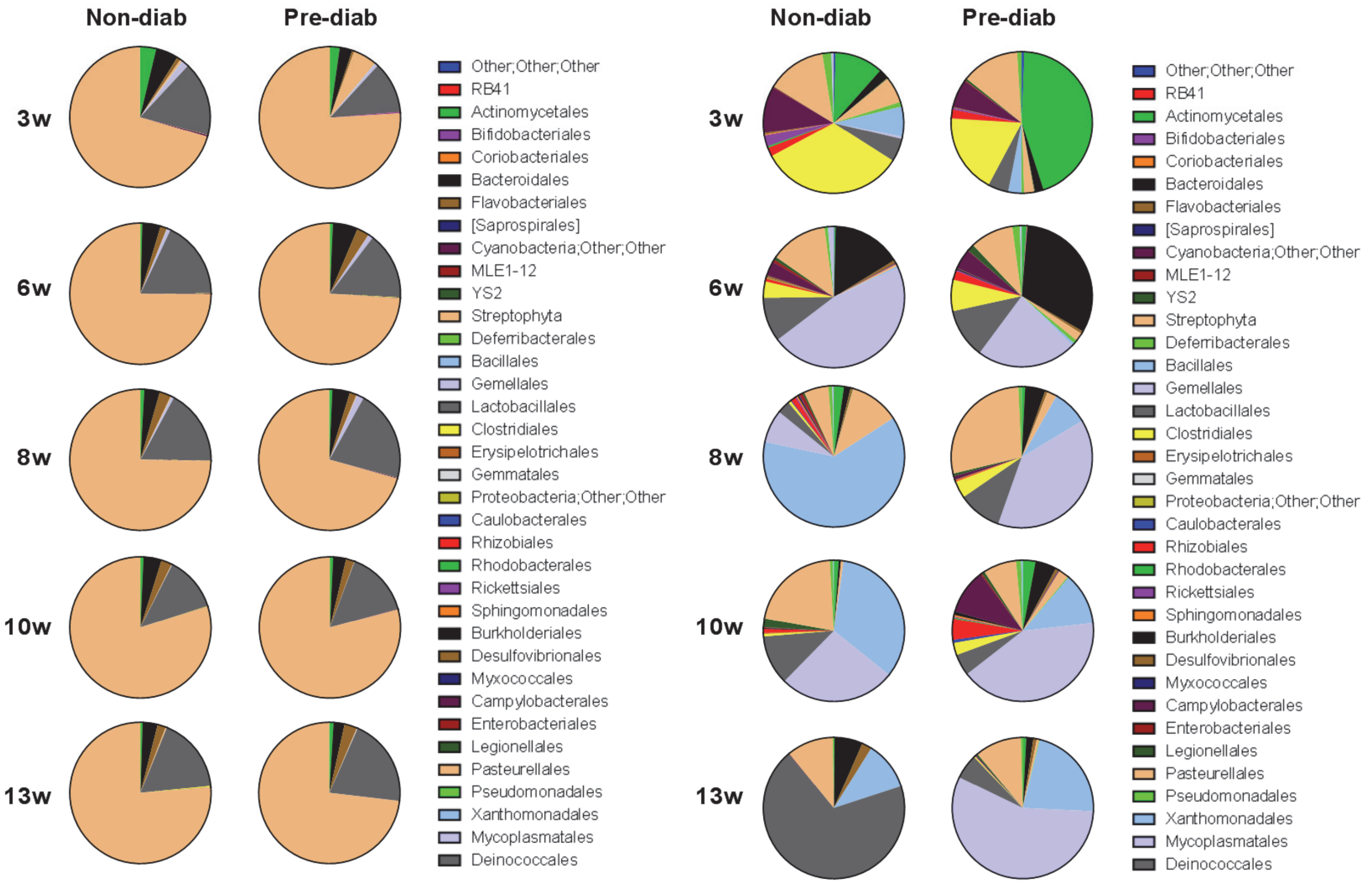

Supplementary Figure 3B: Oral (left two groups) and vaginal (right two groups) microbiota at order level, in non-diab and pre-diab NOD mice at different ages.

Supplementary Figure 3C

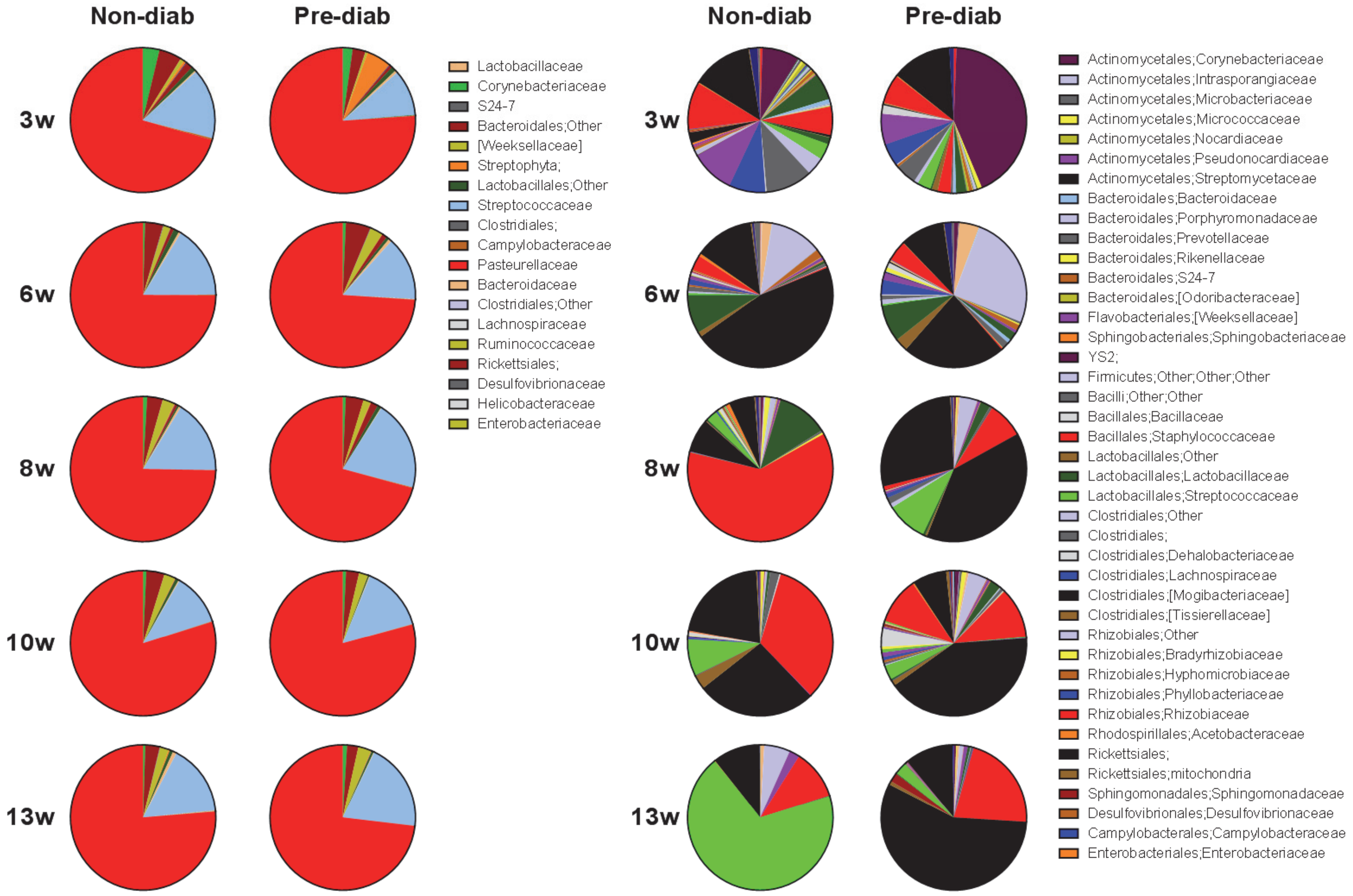

Supplementary Figure 3C: Oral (left two groups) and vaginal (right two groups) microbiota at family level, in non-diab and pre-diab NOD mice at different ages.

Supplementary Figure 3D

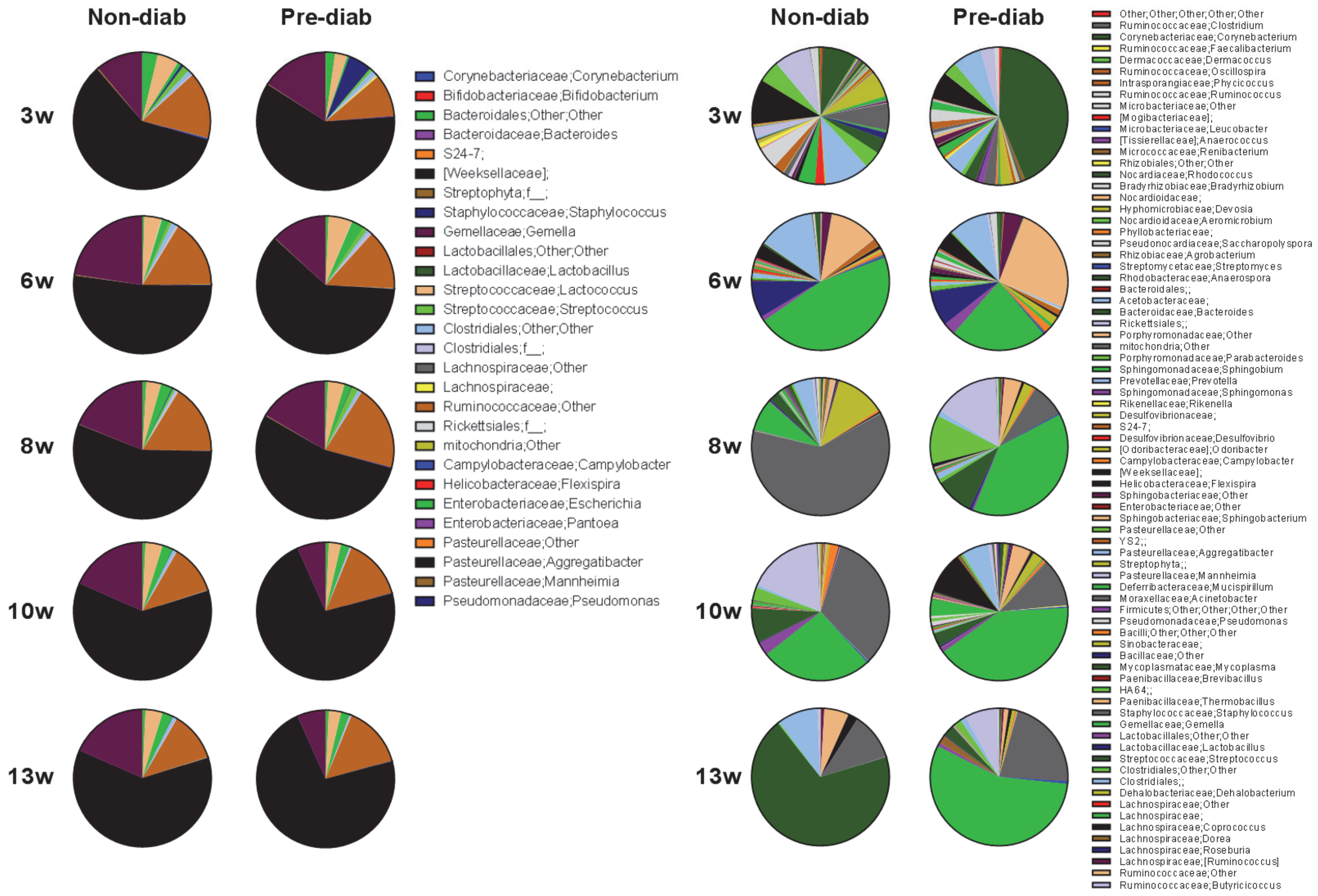

Supplementary Figure 3D: Oral (left two groups) and vaginal (right two groups) microbiota at genus level, in non-diab and pre-diab NOD mice at different ages.

Supplementary Figure 4

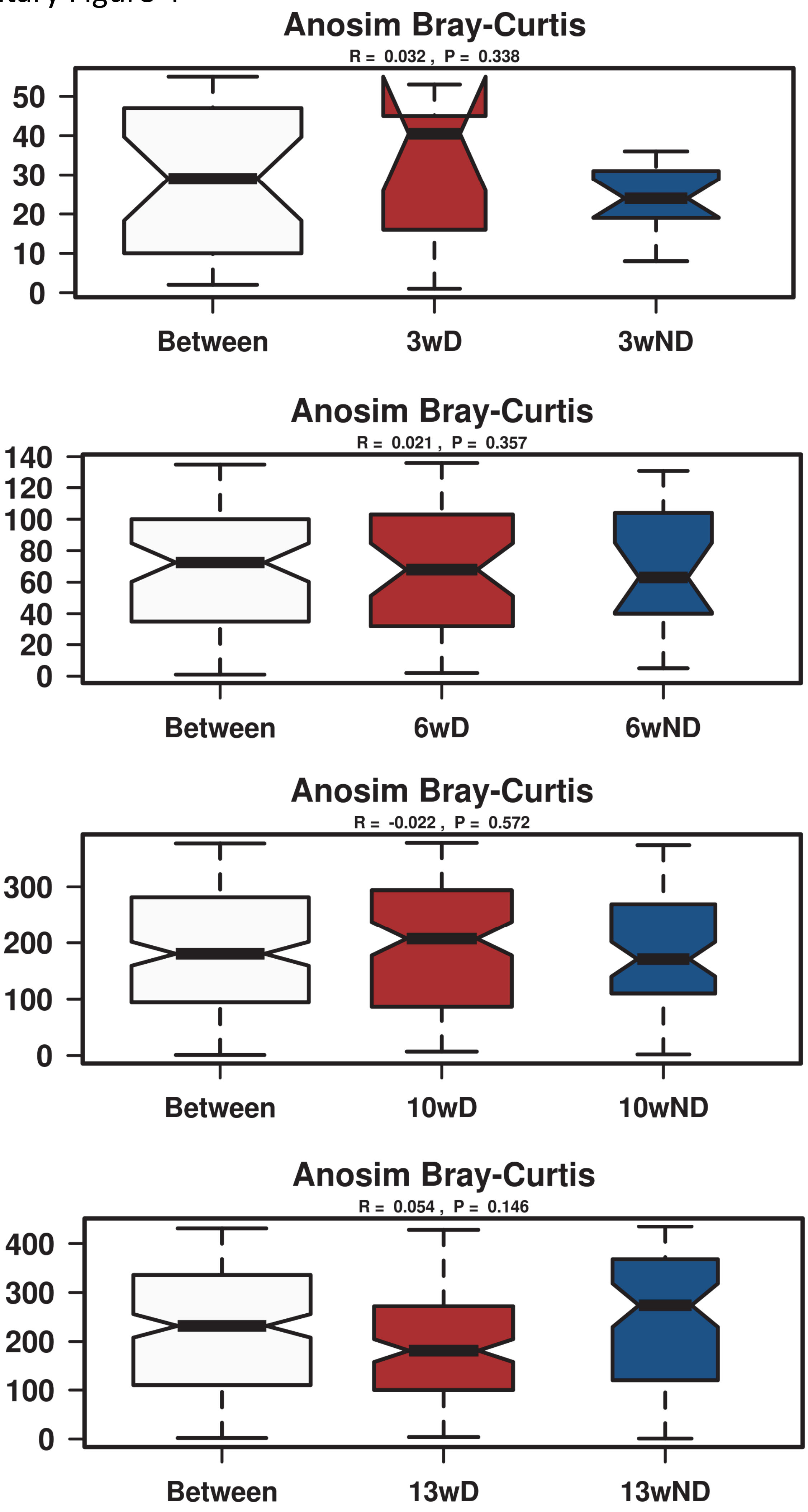

Supplementary Figure 4: Anosim analysis based on Bray-Curtis distance of gut microbiota between non-diabetic (ND) and pre-diabetic (D) NOD mice at 3, 6, 10 and 13 weeks of age.

Supplementary Figure 5

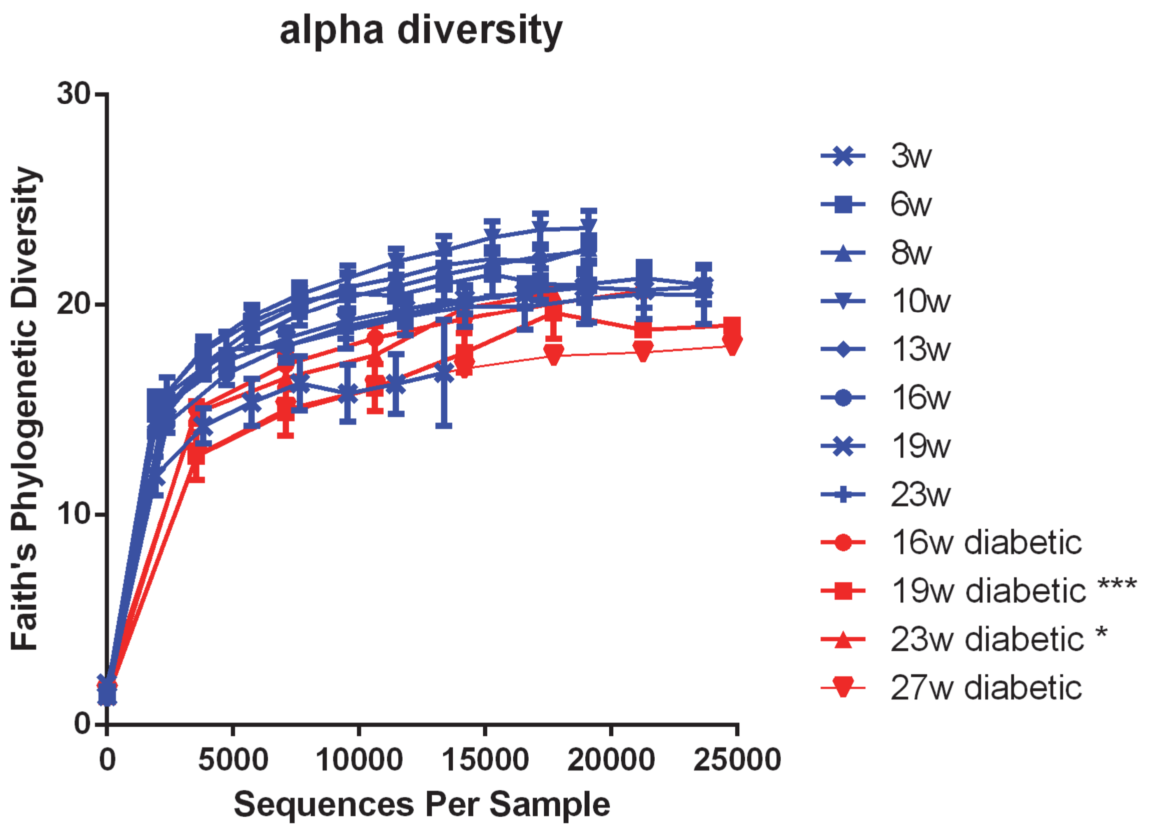

Supplementary Figure 5: Alpha-diversity of gut microbiota of pre-diabetic and diabetic NOD mice at different ages. Diabetic NOD mice at weeks 19 and 23 showed significantly lower diversity than the age-matched non-diabetic counterparts (***, $P<0.001$, *, $P<0.05$ by two way ANOVA).

Supplementary Figure 6

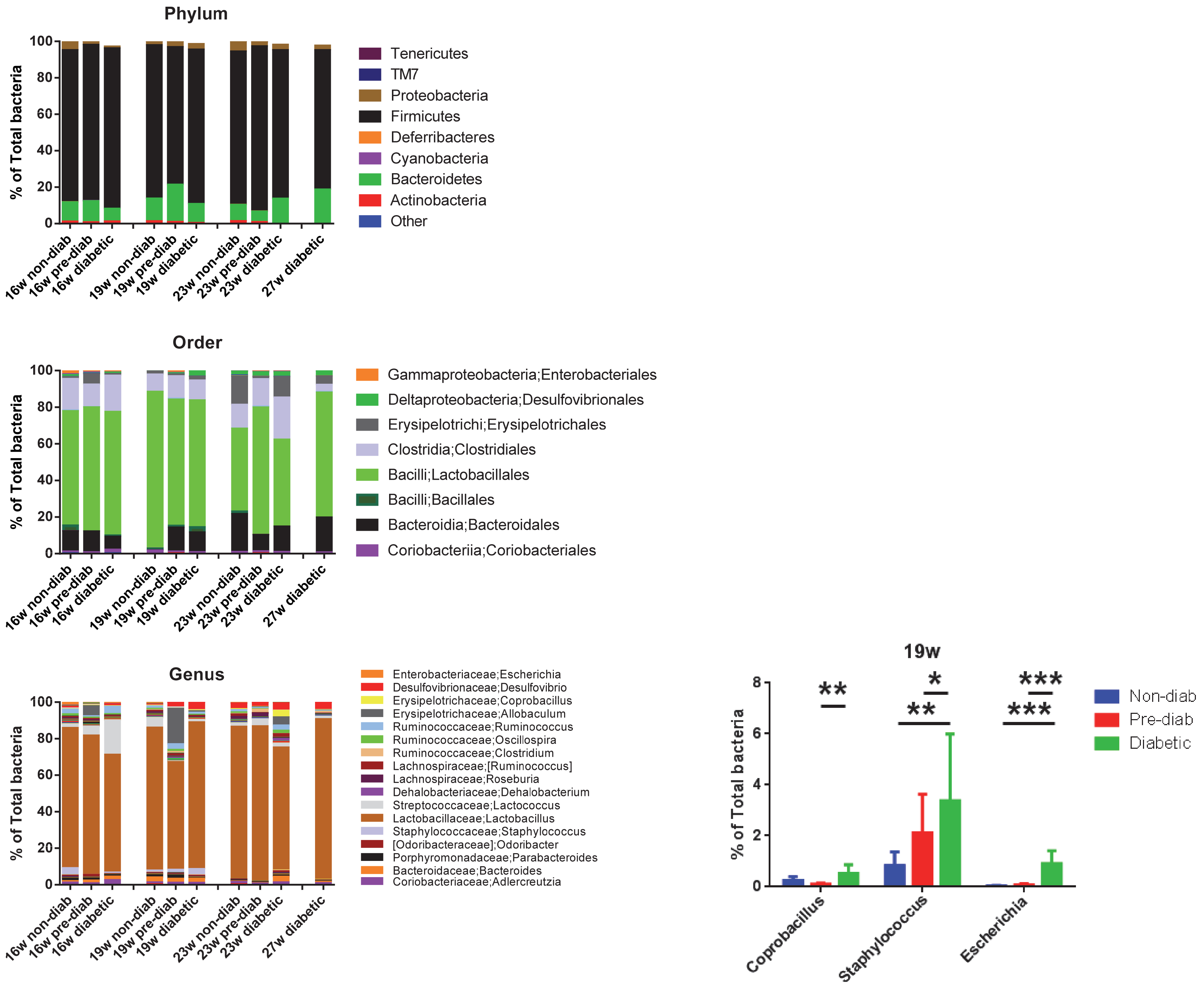

Supplementary Figure 6: Gut bacterial composition of non-diabetic, pre-diabetic and diabetic NOD mice at phylum, order and genus level. The graph shows levels of the genera *Coprobacillus*, *Staphylococcus*, *Escherichia* in mice at 19 weeks of age. (Data are presented as mean +/- SEM and the n=11 each for non-diabetic, pre-diabetic and 5 for diabetic NOD mice. *: P<0.05, **: P<0.01, ***: P<0.001, by multiple t test with Sidak-Bonferroni correction).

Supplementary Figure 7

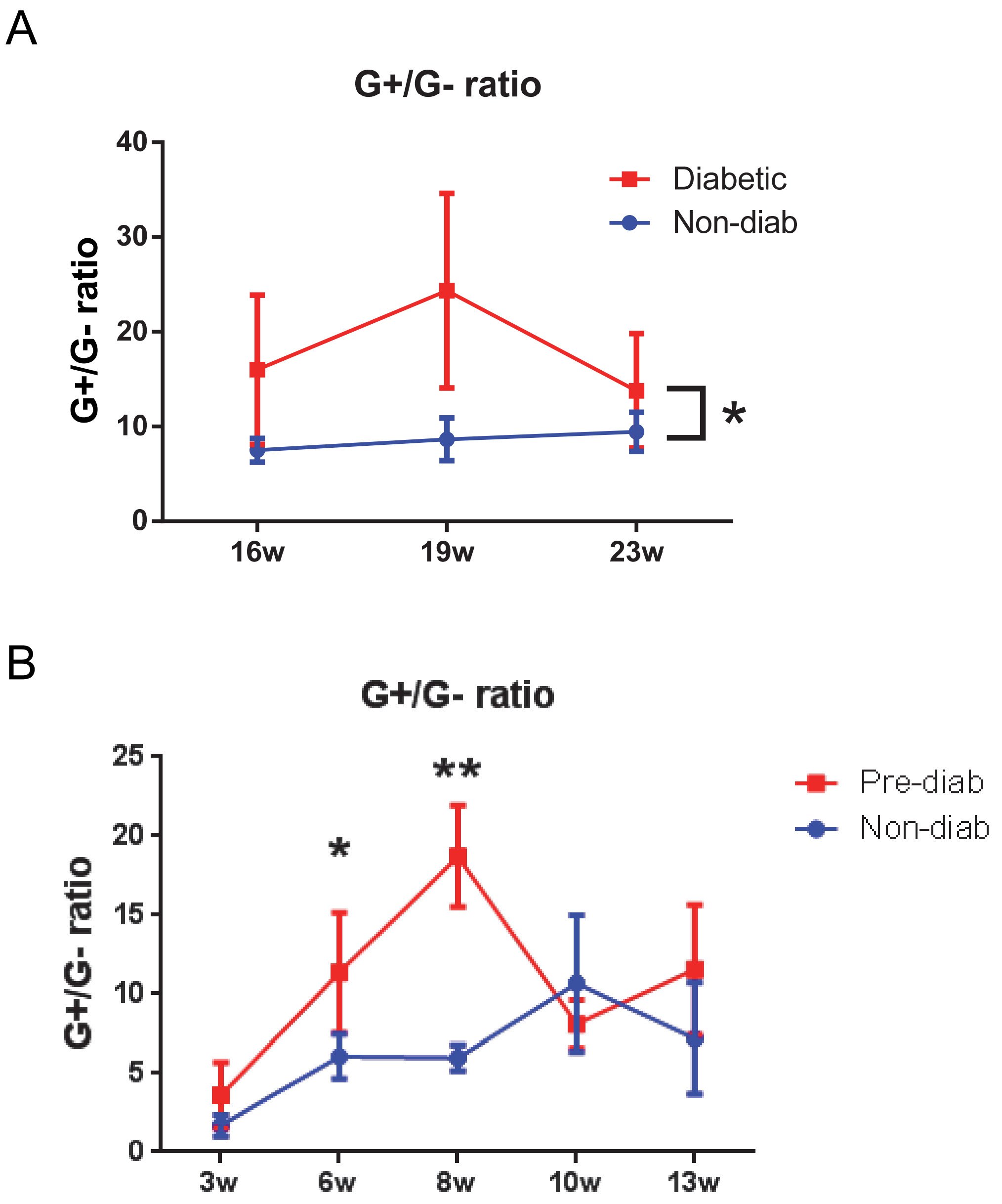

Supplementary Figure 7: A: G+/G- ratios of gut microbiota of non-diabetic and diabetic NOD mice at different ages (*: P=0.0161 by two-way ANOVA). B: G+/G- ratios of gut microbiota of non-diabetic and pre-diabetic NOD mice at different ages (*: P=0.019, **: P=0.001, by multiple t test with Sidak-Bonferroni correction). Data are presented as mean +/- SEM.

Supplementary Figure 8

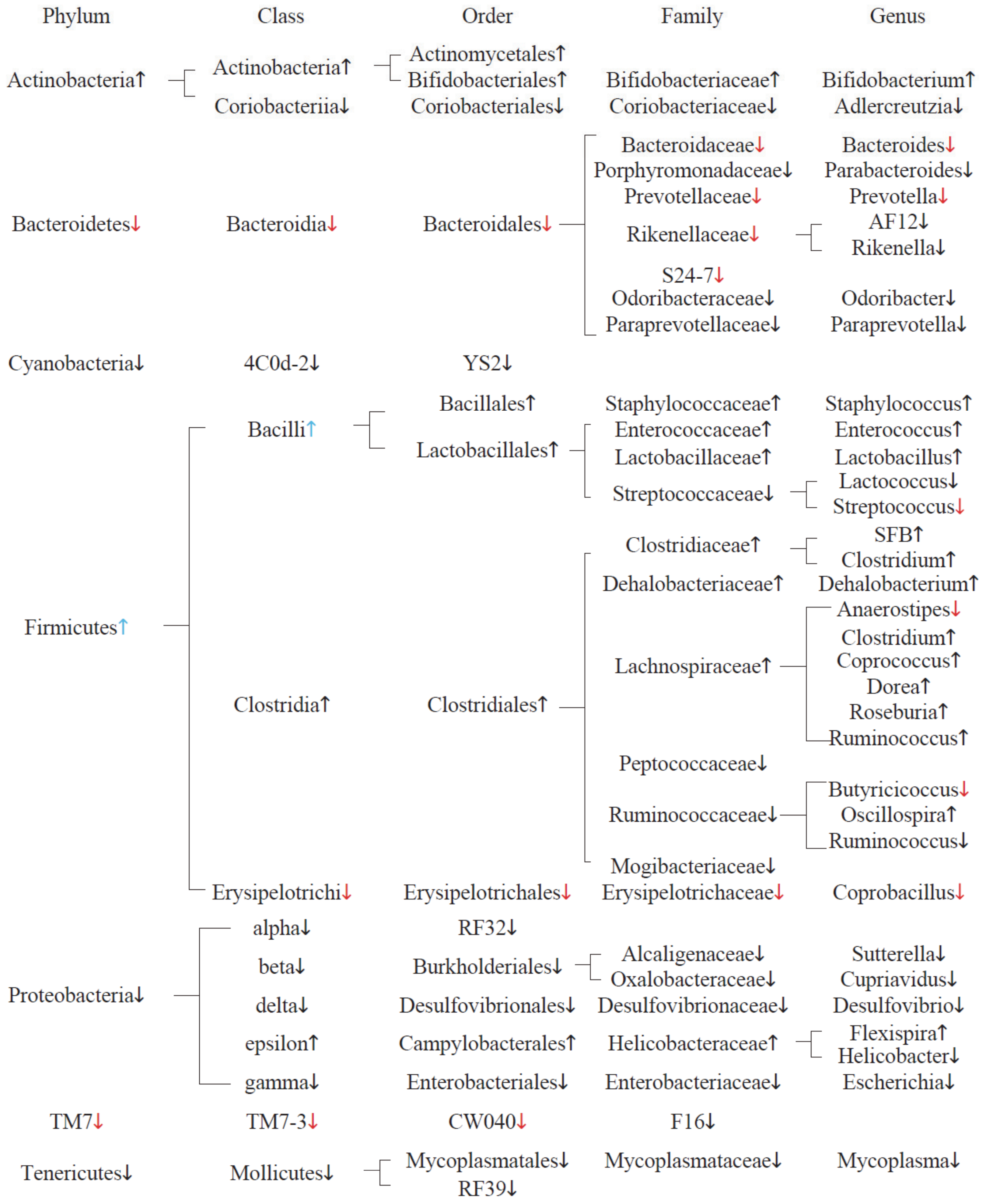

Supplementary Figure 8: Overall taxonomic change of gut microbiota between non-diabetic and pre-diabetic NOD mice at week 8. An upward blue arrow represents a significantly higher trend in pre-diabetic NOD, $P<0.05$ analyzed using multiple t-test corrected by Sidak-Bonferroni method, while a downward red arrow indicates that the average abundance of the taxa was higher in non-diabetic NOD. The black arrows indicate direction of trend that was not statistically significant. $P<0.05$ analyzed using multiple t-test corrected by Sidak-Bonferroni method.

Supplementary Figure 9

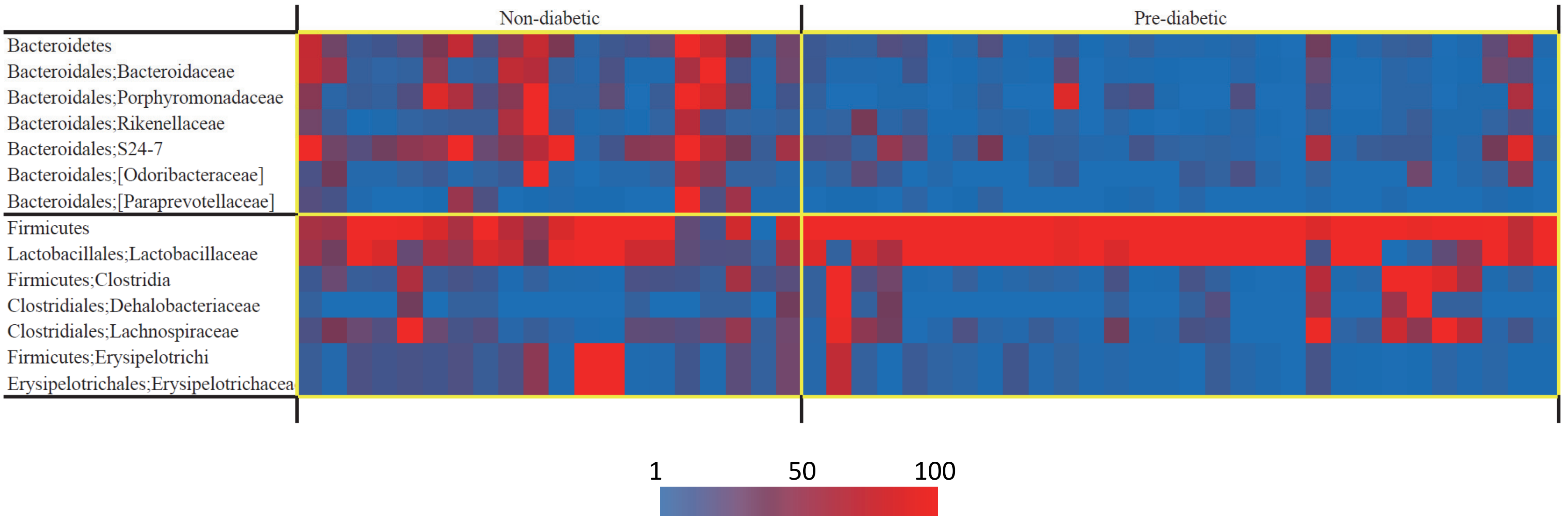

Supplementary Figure 9: Heat map of two phyla (Bacteroidetes and Firmicutes) and some class, order, family taxa differences of gut microbiota in non-diabetic and pre-diabetic NOD mice at week 8. Each column represents a single experimental mouse. The relative abundance of each taxon is scaled to 1-100.

Supplementary Figure 10

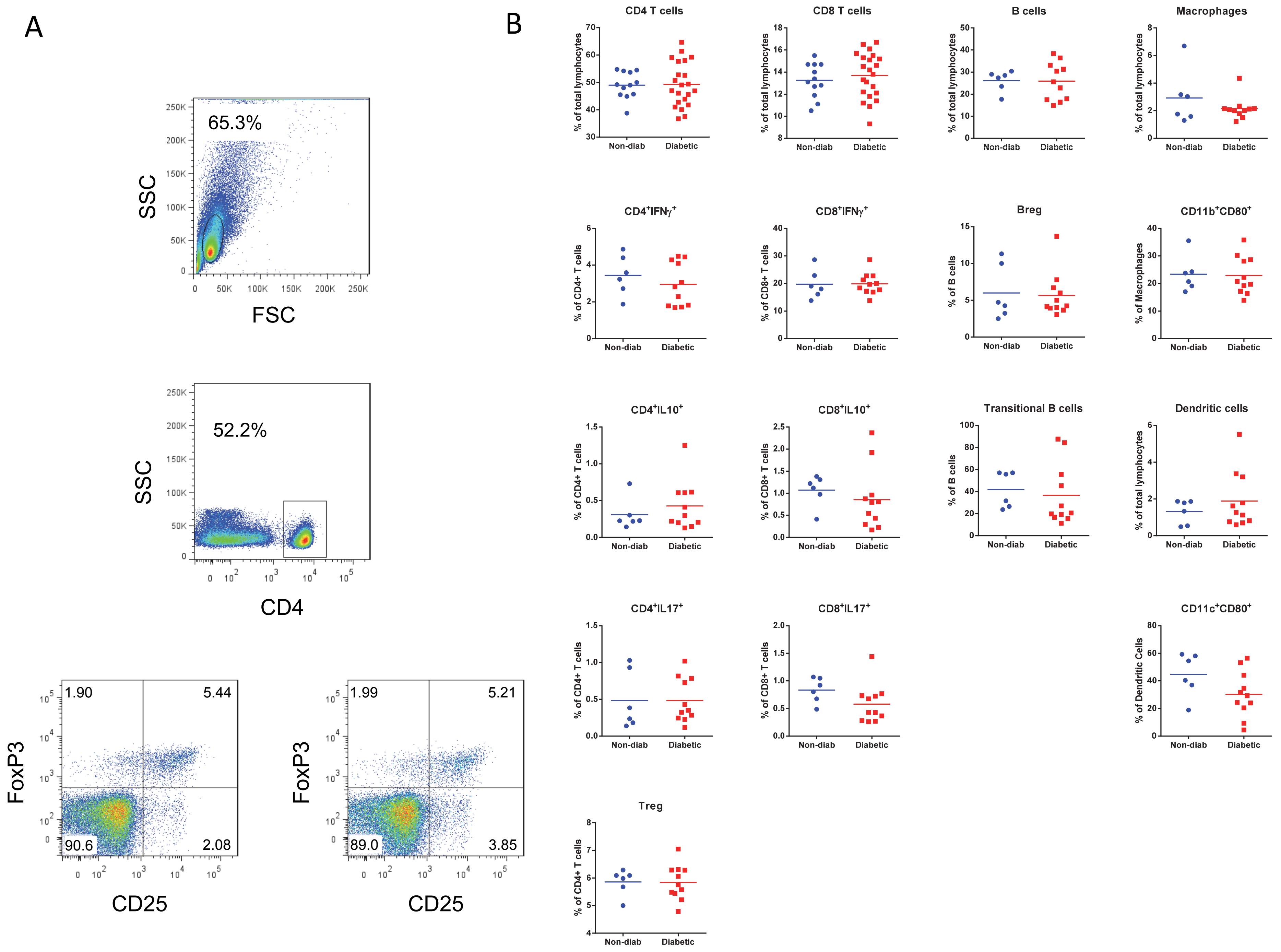

Supplementary Figure 10 : FACS analysis of peripheral blood lymphocytes in NOD mice at week 8. A. Representative gating strategy for Treg (top and middle plots). Bottom plots: non-diab (left:); pre-diab (right). B. Phenotyping of CD4 and CD8 T cells, B cells, Macrophages, Dendritic cells in peripheral blood lymphocytes by flow cytometry. Treg were defined by gated CD4+CD25+FoxP3+ cells, and Breg were defined by gated CD1d^{high}CD5+B220+CD19+ cells.
